# *Klebsiella oxytoca* inhibits *Salmonella* infection through multiple microbiota-context-dependent mechanisms

**DOI:** 10.1038/s41564-024-01710-0

**Published:** 2024-06-11

**Authors:** Lisa Osbelt, Éva d. H. Almási, Marie Wende, Sabine Kienesberger, Alexander Voltz, Till R. Lesker, Uthayakumar Muthukumarasamy, Nele Knischewski, Elke Nordmann, Agata A. Bielecka, María Giralt-Zúñiga, Eugen Kaganovitch, Caroline Kühne, Claas Baier, Michael Pietsch, Mathias Müsken, Marina C. Greweling-Pils, Rolf Breinbauer, Antje Flieger, Dirk Schlüter, Rolf Müller, Marc Erhardt, Ellen L. Zechner, Till Strowig

**Affiliations:** 1 Department of Microbial Immune Regulation, Helmholtz Center for Infection Research, Braunschweig, Germany; 2 ESF International Graduate School on Analysis, Imaging and Modelling of Neuronal and Inflammatory Processes, Otto-von-Guericke University, Magdeburg, Germany; 3 Cluster of Excellence RESIST (EXC 2155), Hannover Medical School, Hannover, Germany; 4 Institute of Molecular Biosciences, University of Graz, BioTechMed-Graz, Graz, Austria; 5 Department Microbial Natural Products, Helmholtz Institute for Pharmaceutical Research Saarland (HIPS), Helmholtz Centre for Infection Research (HZI), Saarbrücken, Germany; 6 Department of Pharmacy, Saarland University, Saarbrücken, Germany; 7 Institute for Biology-Molecular Microbiology, Humboldt-Universität zu Berlin, Berlin, Germany; 8 Institute of Medical Microbiology and Hospital Epidemiology, Hannover Medical School, Hannover, Germany; 9 Division of Enteropathogenic Bacteria and Legionella (FG11)/National Reference Centre for Salmonella and other Bacterial Enteric Pathogens, Robert Koch Institute, Wernigerode, Germany; 10 Central Facility for Microscopy, Helmholtz Centre for Infection Research, Braunschweig, Germany; 11 Mouse-Pathology Platform, Helmholtz Centre for Infection Research, Braunschweig, Germany; 12 BioTechMed-Graz, Institute of Organic Chemistry, Graz University of Technology, Graz, Austria; 13 German Center for Infection Research (DZIF),Partner Site Hannover–Braunschweig, Braunschweig, Germany; 14 Max Planck Unit for the Science of Pathogens, Berlin, Germany; 15 Center for Individualized Infection Medicine, Hannover, Germany

**Keywords:** Microbiome, Bacterial toxins

## Abstract

The *Klebsiella oxytoca* species complex is part of the human microbiome, especially during infancy and childhood. *K. oxytoca* species complex strains can produce enterotoxins, namely, tilimycin and tilivalline, while also contributing to colonization resistance (CR). The relationship between these seemingly contradictory roles is not well understood. Here, by coupling ex vivo assays with CRISPR-mutagenesis and various mouse models, we show that *K. oxytoca* provides CR against *Salmonella* Typhimurium. In vitro, the antimicrobial activity against various *Salmonella* strains depended on tilimycin production and was induced by various simple carbohydrates. In vivo, CR against *Salmonella* depended on toxin production in germ-free mice, while it was largely toxin-independent in mice with residual microbiota. This was linked to the relative levels of toxin-inducing carbohydrates in vivo. Finally, dulcitol utilization was essential for toxin-independent CR in gnotobiotic mice. Together, this demonstrates that nutrient availability is key to both toxin-dependent and substrate-driven competition between *K. oxytoca* and *Salmonella*.

## Abstract (web summary)

*Klebsiella oxytoca* provides protection against *Salmonella* Typhimurium via toxin-dependent inhibition in nutrient-rich, low-competition environments but switches to substrate-driven competition when nutrients are limited.

## Main

A major function of the human microbiota is to provide ‘colonization resistance’ (CR), that is, to prevent or reduce the severity of infections by interfering with pathogen colonization and entry into the underlying host tissue^1^. Diverse mechanisms contribute to CR including competition for essential nutrients^2–4^, metabolites and environmental niches^5,6^ but also the production of inhibitory or toxic compounds^7–12^. Together, they modulate susceptibility towards various enteropathogens such as *Salmonella enterica* serovar Typhimurium (*S*. Typhimurium)^13–15^, multi-drug resistant (MDR) *Klebsiella pneumoniae*^4^ or *Citrobacter rodentium*^8,16^. Members of the *Klebsiella oxytoca* species complex (KoSC), which includes *K. oxytoca*, *Klebsiella* *michiganensis* and *Klebsiella* *grimontii*^17^, were shown to provide CR against their more pathogenic counterparts through overlapping metabolic niches in the gut. Examples include *Klebsiella* sp. providing CR against *Escherichia coli* and *Salmonella*^18^ or communities of enterobacteria that protect against *Salmonella* infection^19^. Moreover, *K. oxytoca* strains conferred CR against MDR *K. pneumoniae* strains through competition for specific beta-glucosidic sugars^4^. While these studies provided mechanistic insight in mouse models, carriage of specific strains of KoSC could also lower the risk for bacteraemia in cancer and haematopoietic cell transplantation patients as well as neonates, providing evidence for beneficial contributions of members of these species in humans^20,21^.

The contribution of KoSC strains to CR might be especially relevant when the prevalence and population densities of these bacteria are high. While only 2–10% of the adult population are natural carriers of KoSC strains, they are prevalent colonizers in infants (10–70%)^4,22–24^. Moreover, while maintaining a relatively low abundance in homeostatic conditions, KoSC members may expand after antibiotic disruption of the microbiota due to the availability of additional metabolic niches. Yet, upon expansion, KoSC strains may also pose a threat to the host due to strain-specific sets of virulence factors including a pathogenicity island (*til* PAI) responsible for the production of the enterotoxic peptides tilivalline (TV) and tilimycin (TM)^22,25–27^. *til* + KoSC strains have been identified as causative agents of a rare condition called antibiotic‐associated haemorrhagic colitis^28–30^ and were also associated with enterocolitis in premature infants^24,31^. In contrast to their well-described effects on mammalian cells^32–34^, the impact on the microbial community remained understudied. Notably, TM, but not TV, also exerts antimicrobial activity against a range of bacteria and promotes mutational evolution in co-resident opportunistic pathogens such as *K. pneumoniae* and *E. coli*^35^. Similarly, *E. coli* strains producing colibactin, another natural genotoxin previously shown to cause DNA mutations in eukaryotic cells, were shown to exert antimicrobial functions and remodel host gut microbiomes^9,36^. Thus, while toxic metabolites and substrate competition between metabolically related Enterobacteriaceae are known to contribute to CR, the ecological contexts in which KoSC members affect the host and the gut ecosystem remain poorly understood.

To better understand how the microbial context influences *K. oxytoca*-mediated restriction of an important enteropathogen, *S*. Typhimurium^37,38^, we studied competition between *K. oxytoca* and *S*. Typhimurium in vitro as well as *S*. Typhimurium-induced enterocolitis in mouse models with disturbed and undisturbed microbiota states. Many but not all tested strains belonging to the KoSC inhibit *S*. Typhimurium in vitro. Genome analysis followed by gene targeting enabled us to identify TM production as a potent inhibitory factor in vitro. Notably, in mouse models, *K. oxytoca* provides CR against *S*. Typhimurium in different microbiota settings via distinct toxin-dependent and -independent mechanisms. The availability of simple carbohydrates links both mechanisms as toxicity predominates with high sugar availability, while microbial competition contributes when carbohydrates are scarce. These findings show that KoSC members can contribute via distinct mechanisms to CR against *S*. Typhimurium.

## Results

### *K. oxytoca* provides CR in various microbiota settings

Antibiotic (abx)-treated specific pathogen-free (SPF) mice and gnotobiotic oligo-mouse microbiota (OMM^12^) mice harbour different densities of *K. oxytoca*^4^ and represent different *K. oxytoca*-colonized community types observed in humans, that is, a high population density of *K. oxytoca* in abx-treated SPF mice and a lower *K. oxytoca* population density in OMM^12^ mice.

**Fig. 1:**
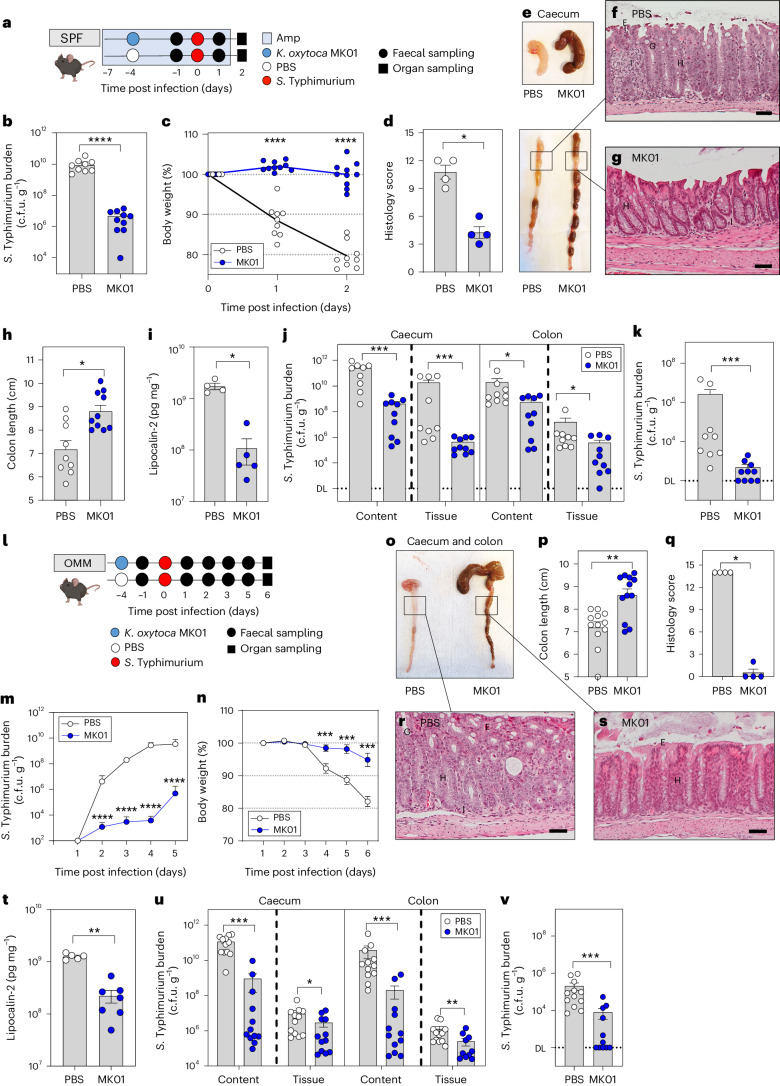
*K. oxytoca* provides CR in different microbiota settings. **a**, Ampicillin-treated SPF mice were colonized with *K. oxytoca* MK01 or left untreated 4 days before infection. On day 0, mice were orally infected with *S*. Typhimurium. BWL and faecal colonization were monitored until day 2 p.i. when organs were sampled for examination of *S*. Typhimurium burden. Amp, ampicillin. **b**,**c**, *S*. Typhimurium burden in the faeces on day 1 p.i. (**b**) and resulting BWL (**c**). **d**, Histological inflammation score of the proximal colon. **e**, Organ morphology of caecum and colon. **f**,**g**, Representative pictures of HE-stained sections of the proximal colon for both groups: PBS (**f**) and MK01 (**g**). E, erosion; G, goblet cells; H, hyperplasia; I, inflammatory cells. Scale bars, approximately 50 μm. **h**,**i**, Colon length (**h**) and lipocalin-2 levels (**i**) in the colon content on day 2 p.i. Mean ± s.e.m. of one experiment with *n* = 4 (PBS) or *n* = 5 (WT) mice. **j**, *S*. Typhimurium burden in the caecum and colon. DL, level of detection. **k**, *S*. Typhimurium burden in the liver. In **b**, **c**, **h**, **j** and **k**, mean ± s.e.m. of three pooled experiments with *n* = 9 (PBS) and 10 (MK01) mice. **l**, OMM^12^ mice were colonized with *K. oxytoca* MK01 or left untreated 4 days before infection. On day 0, mice were orally infected with *S*. Typhimurium, and BWL and faecal colonization were monitored until day 6 p.i. when organs were sampled for examination of *S*. Typhimurium burden. **m**,**n**, Faecal burden of *S*. Typhimurium (**m**) and resulting BWL over the time of infection (**n**). **o**–**s**, Visual examination of caecum and colon morphology (**o**) with resulting colon length (**p**) and histological inflammation score of the proximal colon (**q**), with representative HE-stained sections of both groups: PBS (**r**) and MK01 (**s**). Scale bars, 50 μm. **t**, Lipocalin-2 levels in the colon content on day 6 p.i., mean ± s.e.m. of two experiments with *n* = 5 (PBS) or *n* = 7 (WT) mice. **u**, *S*. Typhimurium burden in the caecum and colon. **v**, *S*. Typhimurium burden in the liver. In **m**, **n** and **p**–**v**, mean ± s.e.m. of *n* = 3 experiments with *n* = 12 mice per group. In **d** and **q**, mean ± s.e.m. of one experiment with *n* = 4 mice. In **b**–**d**, **h**–**k**, **m**, **n**, **p**, **q** and **t**–**v**, two-tailed Mann–Whitney *U*-test with *P < 0.05, **P < 0.01, ***P < 0.001 and ****P < 0.0001. See also Extended Data Figs. 1 and 2. Source data

**Extended Data Fig. 1:**
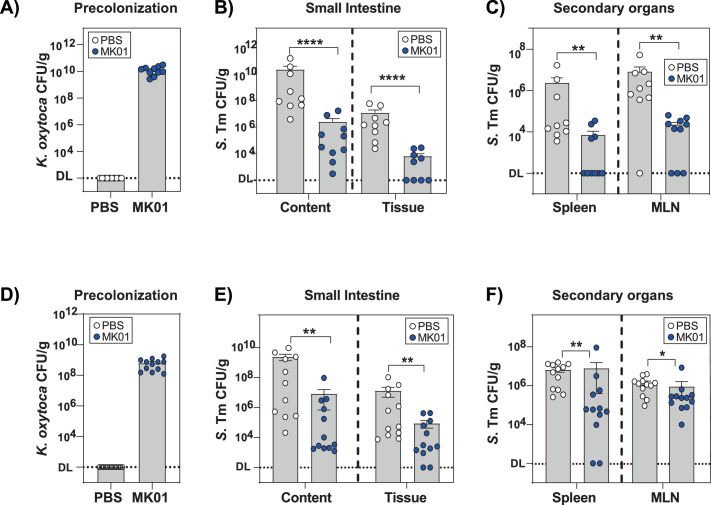
*K. oxytoca* provides colonization resistance in different microbiota settings. (**a**) Fecal colonization levels of *K. oxytoca* MK01 precolonized SPF mice at day 0. (**b**) *S*. Tm burden in the lumen and tissue of small intestine and (**c**) various secondary organs including spleen and MLN of *S*. Tm-infected SPF mice at day 2 p.i. (**a-c**) Mean ± SEM of three independent experiments with n = 9 (PBS) or 10 (MK01) mice per group are displayed. P-values indicated represent two-tailed Mann-Whitney U-test with *p < 0.05, **p < 0.01, ***p < 0.001 and ****p < 0.0001. (**d**) Fecal colonization levels of *K. oxytoca* MK01 precolonized OMM^12^ mice at day 0. (**e**) *S*. Tm burden in the lumen and tissue of small intestine and (**f**) various secondary organs including spleen and MLN of *S*. Tm infected OMM^12^ mice at day 6 p.i. (**d-f**) Mean ± SEM of three independent experiments with n = 12 mice per group are displayed. P-values indicated represent two-tailed Mann-Whitney U-test with *p < 0.05, **p < 0.01, ***p < 0.001 and ****p < 0.0001. Source data

To create an antibiotic-disturbed gut environment, mice were treated with ampicillin. A subset of animals was then precolonized with the ampicillin-resistant *K. oxytoca* MK01 strain resulting in high population densities (mean = 1.33 × 10^10^ c.f.u. (colony-forming units) per g faeces; Extended Data Fig. 1a). Four days later, both groups of mice were infected with *S*. Typhimurium EM12442, an SL1344 strain expressing a chromosomal ampicillin resistance (Fig. 1a). Notably, *K. oxytoca* reduced *S*. Typhimurium colonization (Fig. 1b) and intestinal inflammation. Specifically, *K. oxytoca* attenuated body weight loss (BWL) (Fig. 1c) and reduced inflammation in precolonized mice, as indicated by macroscopic inspection of intestinal organ morphology, colon length and histological scoring of colon tissue sections (Fig. 1d–h). Concomitantly, levels of lipocalin-2 were reduced in *K. oxytoca*-precolonized compared to control mice (Fig. 1i). Finally, colonization of *S*. Typhimurium was reduced not only in intestinal content and tissues (10- to 10^2^-fold) and lumen (10^2^- to 10^3^-fold) in the abx-treated SPF model but also in the liver, demonstrating that *K. oxytoca* also reduced *S*. Typhimurium translocation (Fig. 1j,k and Extended Data Fig. 1b,c).

Next, the impact of *K. oxytoca* on *S*. Typhimurium infection was investigated in OMM^12^ mice, which lack natural CR against *S*. Typhimurium circumventing the need for antibiotic pre-treatment. One group of OMM^12^ mice was precolonized with *K. oxytoca* MK01 for 4 days before all mice were infected subsequently with *S*. Typhimurium (Fig. 1l). OMM^12^ mice showed stable colonization of *K. oxytoca* (mean = 6.4 × 10^8^ c.f.u. per g faeces) at 10^2^-fold lower levels than observed in abx-treated SPF mice (Extended Data Fig. 1a,d). Disease development in this model was delayed compared to the SPF-abx model, and *S*. Typhimurium-infected mice displayed signs of severe inflammation only on day 6 post infection (p.i.) in line with previous studies^39,40^. *K. oxytoca*-colonized OMM^12^ mice showed decreased faecal loads of *S*. Typhimurium (Fig. 1m), attenuated BWL (Fig. 1n), reduced *S*. Typhimurium-induced disease as quantified by macroscopic examination of the tissue, colon length, histological scores (Fig. 1o–s), lipocalin-2 quantification (Fig. 1t) and reduced colony-forming units along the gastrointestinal tract and extra-intestinal organs (Fig. 1u,v and Extended Data Fig. 1e,f). Together, these results show that *K. oxytoca* MK01 confers CR against *S*. Typhimurium in two distinct mouse models despite 100-fold differences in population density.

### KoSC strains vary regarding their inhibitory capabilities

**Fig. 2:**
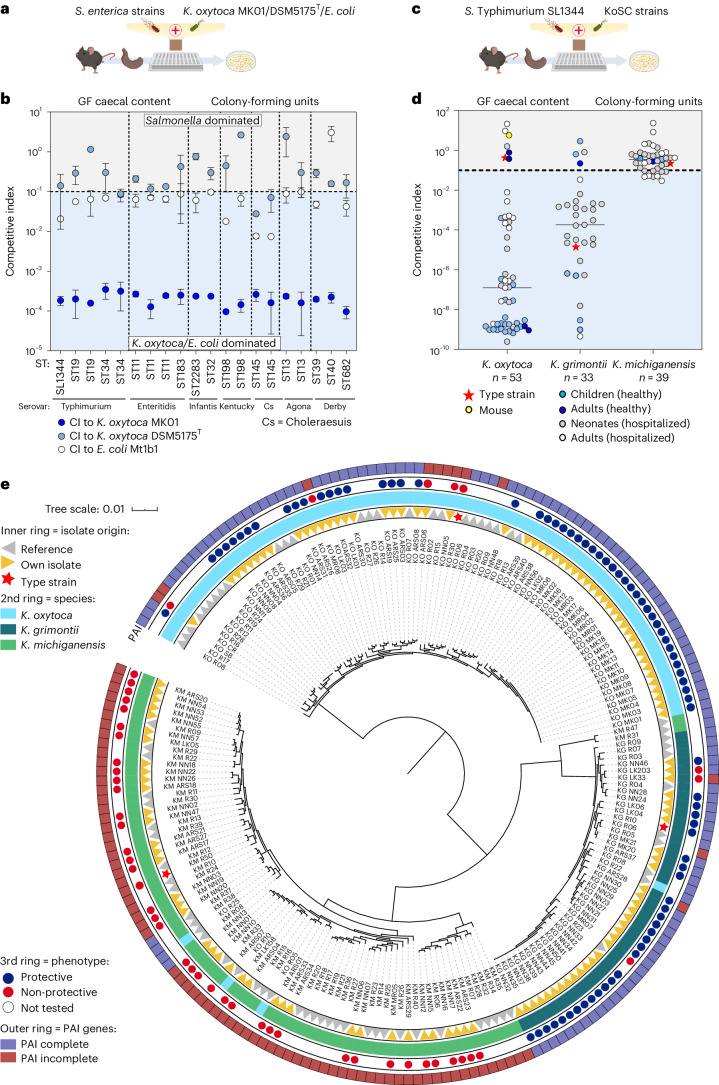
Broad protective capability is shared among many *K. oxytoca* and *K. grimontii* but not *K. michiganensis* strains. **a**, Aerobic co-cultures of different *S. enterica* serovars (Typhimurium, Enteritidis, Infantis, Kentucky, Choleraesuis, Agona and Derby) with *K. oxytoca* MK01/DSM5175^T^ or *E. coli* Mt1b1 in a 1:10 ratio in isolated GF caecal content. **b**, Resulting CI of various *S. enterica* serovars against *K. oxytoca* MK01, DSM5175^T^ and *E. coli* Mt1b1. The horizontal dashed line indicates the starting ratio of bacteria (index = 0.1). The mean ± s.e.m. of *n* = 2 biological experiments with *n* = 2 technical replicates are displayed. ST, sequence type. **c**, *S*. Typhimurium was co-cultivated with different KoSC strains from different origins as described before. **d**, Resulting CI of *S*. Typhimurium colony-forming units in co-cultures with various strains from the KoSC species, *K. oxytoca*, *K. michiganensis* and *K. grimontii*. The dashed line indicates the starting ratio of bacteria (index = 0.1). The different colours of each dot represent the origin of the KoSC isolate in each co-culture. Each dot represents the mean of *n* = 2–3 independent experiments with *n* = 2–3 technical replicates. **e**, Phylogenetic distribution of the 122 KoSC isolates in comparison with 75 publicly available genomes including three type strains depicted in the inner ring. The species identity of the strains (second inner ring), the phenotype against *S*. Typhimurium in the ex vivo co-culture screening (third ring) and their names are indicated. The tree is constructed based on the sequence variations within 712 core genes using fasttree. See also Extended Data Fig. 3. Source data

**Extended Data Fig. 2:**
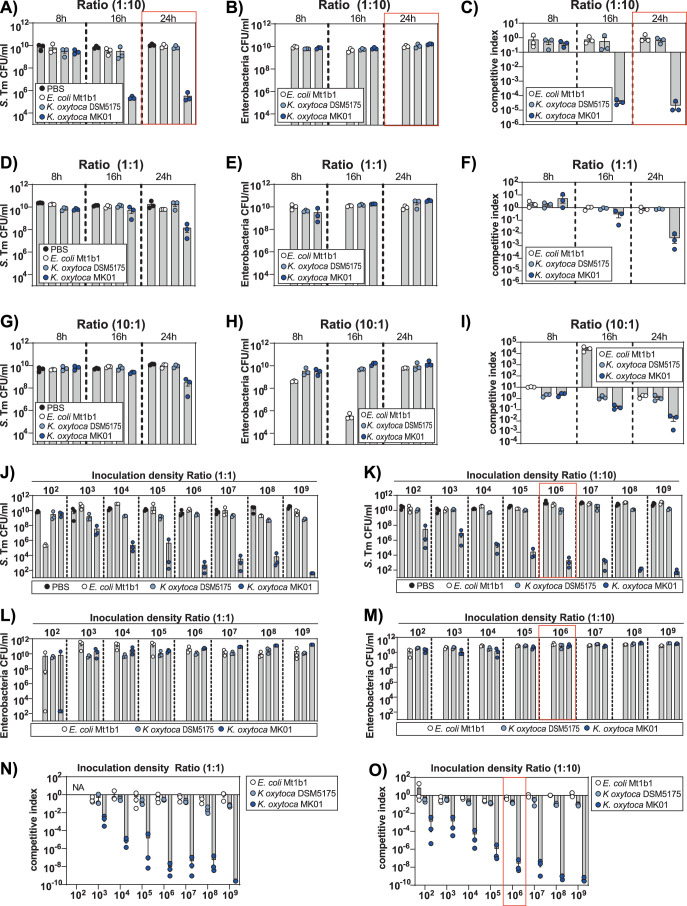
*K. oxytoca* MK01 inhibits *S*. Tm better than two reference bacteria. Aerobic co-cultures of *S*. Tm with Enterobacteria (*K. oxytoca* MK01, *K. oxytoca* DSM5175^T^ or *E. coli* Mt1b1) in in germfree cecal content with varying ratios and incubation times. Resulting CFUs of (**a**) *S*. Tm and (**b**) Enterobacteria in a 1:10 ratio and (**c**) corresponding competitive index (CI). Resulting CFUs of (**d**) *S*. Tm and (**e**) Enterobacteria in a 1:1 ratio and (**f**) corresponding CI. (**g**) Resulting CFUs of *S*. Tm and (**h**) Enterobacteria in a 10:1 ratio and (**I**) corresponding CI. **(j-o**) Titration of various inoculation densities in a 1:1 and 1:10 ratio for *S*. Tm. vs. *K. oxytoca* MK01, *K. oxytoca* DSM5175^T^ and *E. coli* Mt1b1 after 24 h of aerobic cultivation. (**j**) Resulting CFUs of various inoculation densities of *S*. Tm in a 1:1 and (**k**) 1:10 starting ratio. (**l**) Resulting CFUs of various inoculation densities of the enterobacterial strains in a 1:1 and (**m**) 1:10 starting ratio. (**n**) Corresponding CI of both bacteria at various inoculation densities and a starting ratio of 1:1 or (**o**) 1:10. (**a-n**) The mean ± SEM of one experiment with n = 3 technical replicates is displayed. Source data

Next, we utilized a simplified screening system previously established to evaluate interspecies competition ex vivo^4^ (Fig. 2a). In brief, the caecal content of germ-free (GF) mice was used as a media base representing substrate availability in the disturbed gut. Initially, we tested various pathogen to commensal inoculation ratios, incubation times and inoculation densities (Extended Data Fig. 2a–o). To compare the inhibitory properties of *K. oxytoca* MK01 to other Enterobacteria, we included the type strain of *K. oxytoca* (DSM5175^T^) as well as an *E. coli* strain (Mt1b1) with known capacity to compete with *S*. Typhimurium in OMM^12^ mice^41^. To compare the interaction of the different Enterobacteria, we calculated a competitive index (CI) ratio for each bacterial co-culture (see Methods section for details). We observed a pronounced and stable reduction of *S*. Typhimurium growth mediated by *K. oxytoca* MK01, but not *K. oxytoca* DSM5175^T^ or *E. coli* Mt1b1 with a 1:10 pathogen to commensal ratio and 24 h of incubation (Extended Data Fig. 2a–c). It is noteworthy that, for the *K. oxytoca* MK01-mediated inhibition of *S*. Typhimurium, an inoculation density-dependent effect was identified. Thus, for subsequent analyses, we continued with 24 h of incubation, a 1:10 pathogen to commensal ratio, and 10^6^ c.f.u. as inoculation density to sustain a robust phenotype.

**Extended Data Fig. 3:**
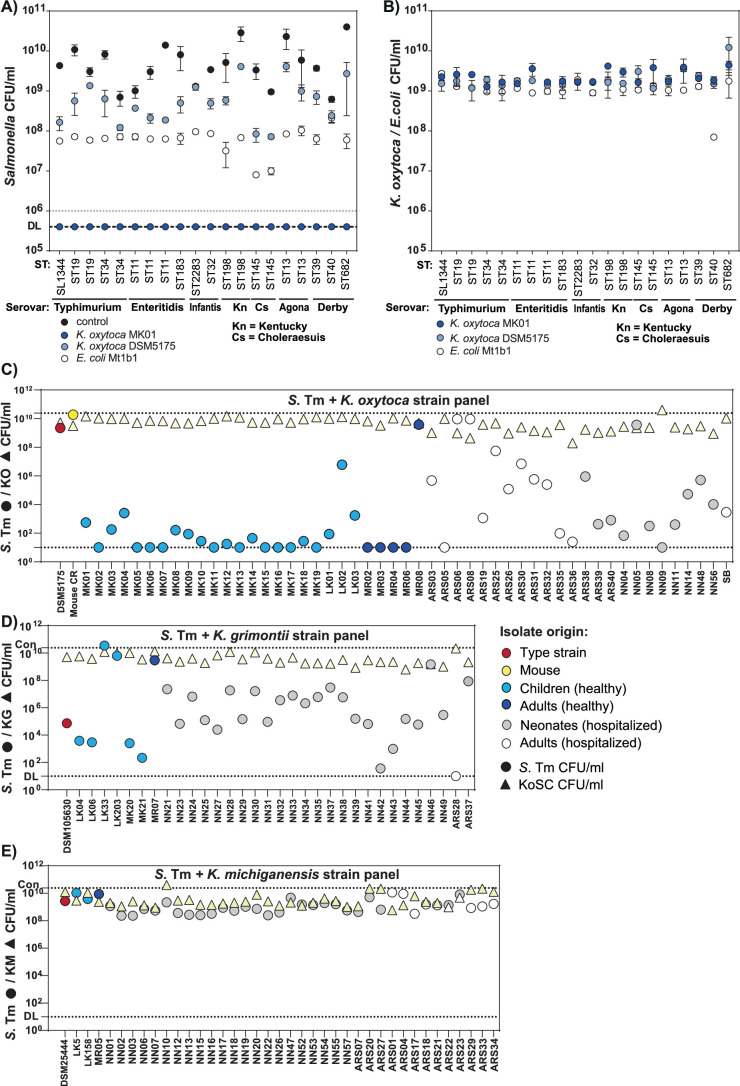
Broad protective capability is shared among many *K. oxytoca* and *K. grimontii* but not *K. michiganensis* strains from different origins. (**a**) *S. enterica* and (**b**) Enterobacteria (*K. oxytoca* MK01/DSM5175^T^, *E. coli* Mt1b1) CFUs after 24 h of co-cultivation. Dashed line indicates level of detection (DL). (**a-b**) The mean ± SEM of two independent biological experiments with n = 2 technical replicates are displayed. (**c**) Resulting *S*. Tm (dots) and KoSC (triangles) CFUs of co-cultures with various strains from the KoSC, including *K. oxytoca* = KO, (**d**) *K. grimontii* = KG and (**e**) *K. michiganensis* = KM. The different colors of each dot represent the origin of the KoSC isolate in each co-culture (type strains = red star, mouse gut isolate = yellow dot, healthy children = light blue dot, healthy adults = dark blue dot, neonatal isolate from medical environment = light grey dot and adult isolate from medical environment = white dot). Dots represent the levels of *S*. Tm in each co-culture, while triangles represent the corresponding CFUs of the KoSC strain in each condition. (**c-e**) The mean of 2 (*K. grimontii*) or 3 (*K. oxytoca / K. michiganensis* panel) independent biological experiments with n = 2 technical replicates are displayed. Source data

We next asked whether *K. oxytoca-*mediated inhibition of *S*. Typhimurium was limited to strain EM12442 used for the infection experiments and compared a clinical strain panel of different serovars and multilocus sequence types of *S. enterica* (Fig. 2b and Supplementary Table 1). All of the *Salmonella* strains were unaffected or inhibited ≤10-fold in co-culture with *E. coli* Mt1b1 or *K. oxytoca* DSM5175, while all *Salmonella* strains fell below the detection limit after co-culture with *K. oxytoca* MK01 (Extended Data Fig. 3a–c). Thus, *K. oxytoca* MK01 inhibited not only *S*. Typhimurium EM12442 but also various clinically relevant *Salmonella* serovars in vitro more efficiently than two reference bacterial strains.

Next, we tested whether this inhibitory capacity was unique to *K. oxytoca* MK01 or shared by other members of the KoSC. Therefore, diverse KoSC strains of different, mainly human, origins were co-cultured with *S*. Typhimurium (Fig. 2c and Supplementary Table 1). It is interesting that the inhibitory properties varied according to species (Fig. 2d and Extended Data Fig. 3c–e). While the majority of *K. oxytoca* (46 of 53, 86.7%) and *K. grimontii* (29 of 33, 87.9%) strains suppressed *S*. Typhimurium >10-fold (CI < 0.01), none of the *K. michiganensis* strains (0 of 39) mediated this level of inhibition. Importantly, *Klebsiella* strains grew to comparable levels in co-cultures independent of their ability to inhibit *S*. Typhimurium (Extended Data Fig. 3c–e). In summary, most *K. oxytoca* and *K. grimontii* but not *K. michiganensis* strains inhibited *S*. Typhimurium growth in vitro independent of origin.

### The *til* PAI correlates with inhibition

To identify genetic differences between inhibitory and non-inhibitory KoSC strains, they were whole-genome sequenced and annotated (*n* = 122). A phylogenetic tree was generated based on the tested KoSC strains and 75 publicly available genomes including the three species’ type strains (KOR30 = DSM5175^T^, KGR10 = DSM105630^T^ and KMR50 = DSM25444^T^) (Fig. 2e and Supplementary Table 1). *K. oxytoca* and *K. grimontii* strains do not appear to cluster distinctively based on the inhibitory properties, but this is difficult to evaluate because the number of strains without inhibitory properties is low. Nevertheless, the inhibitory phenotype coincided in almost all strains with the presence of the *til* PAI (Supplementary Table 1). Generally, strains encoding none or not all essential genes of the *til* PAI were less protective than those encoding a complete *til* PAI. Only 4 of 125 analysed strains (*K. grimontii* LK203 and MR07, *K. oxytoca* ARS06 and ARS08) failed to protect against *S*. Typhimurium despite carrying all essential *til* genes (Fig. 2e and Supplementary Table 1).

### *K. oxytoca* toxin production is essential for the inhibition

**Fig. 3:**
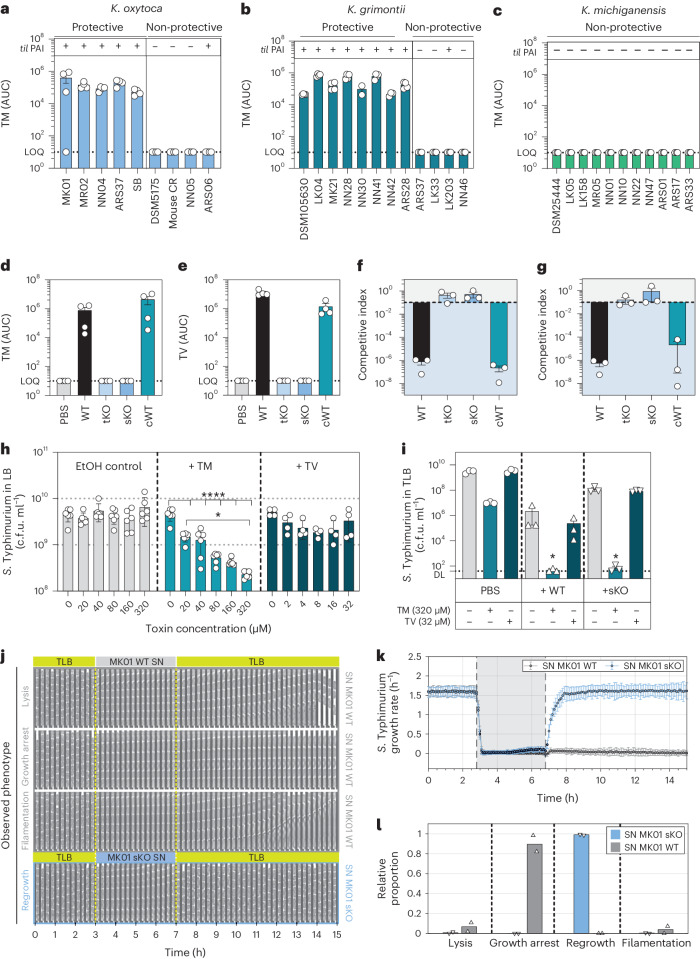
*K. oxytoca* toxin production is crucial for protective capability in vitro. SNs of various KoSC strains were assessed for TM (*m*/*z* = 235) and TV (*m*/*z* = 334) using HPLC. **a**–**c**, Resulting AUC for TM in sample SNs of *K. oxytoca* (**a**), *K. grimontii* (**b**) and *K. michiganensis* (**c**) strains. Mean ± s.e.m. of one experiment with *n* = 4 cultures. LOQ, limit of quantification. **d**,**e**, AUC for TM (**d**) and TV (**e**) of *K. oxytoca* MK01 WT, tKO, sKO and complemented mutant (cWT). Mean ± s.e.m. of one experiment with *n* = 4 cultures. **f**,**g**, CI of *S*. Typhimurium after 24 h of co-cultivation with *K. oxytoca* WT and mutants in GF caecal content media (**f**) and TLB (**g**). The dashed line indicates the starting ratio of bacteria (index = 0.1). Mean ± s.e.m. of *n* = 3 independent experiments with *n* = 2–3 technical replicates per group. **h**, colony-forming units of *S*. Typhimurium grown for 24 h in LB supplemented with various concentrations of TM/TV or EtOH as solvent control. Mean ± s.e.m. of two experiments with *n* = 4–6 independent cultures of *S*. Typhimurium. *P* values indicated represent one-way ANOVA with Tukey’s multiple comparisons test with *P < 0.05 and ****P < 0.0001. **i**, Resulting *S*. Typhimurium colony-forming units after co-cultivation with MK01 WT and sKO strain in the absence or presence of 320 µM TM or 32 µM TV. Mean ± s.e.m. of *n* = 3 experiments with *n* = 2 technical replicates. *P* values indicated represent one-way ANOVA with Dunn’s multiple comparisons test with *P < 0.05. **j**, Representative kymographs of *S*. Typhimurium mother lineages upon 4 h exposure to spent SN from MK01 WT or MK01 sKO displaying cell lysis, growth arrest or filamentation or regrowth. **k**, *S*. Typhimurium growth rates over time. Mean ± s.d. of 1 out of 3 mother machine experiments with *n* = 36,970 cell division cycles. **l**, The relative proportion of mother lineages corresponding to different phenotypes upon exposure to SN of MK01 WT or MK01 sKO. Mean from *n* = 2 independent experiments, corresponding to *n* = 552 mother cells. See also Extended Data Figs. 4 and 5. Source data

**Extended Data Fig. 4:**
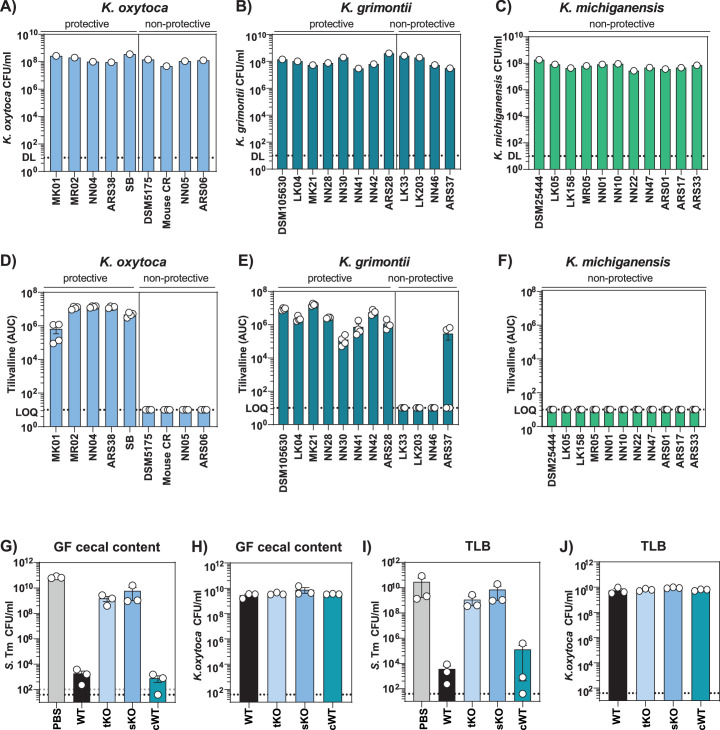
*K. oxytoca* toxin production is crucial for protective capability against *Salmonella* ex vivo*.* (**a**) CFUs of mono-cultures of various protective and non-protective *K. oxytoca* and (**b**) *K. grimontii* strains as well as (**c**) a representative panel of non-protective *K. michiganensis* strains in TLB medium. (**a-c**) CFUs of one culture are displayed. (**d**) Resulting area under curve (AUC) for TV in sample supernatants of different protective and non-protective isolates of *K. oxytoca*, (**e**) *K. grimontii* and (**f**) non-protective *K. michiganensis* strains. (**d-f**) Results represent mean ± SEM of n = 4 independent biological cultures. (G-H) *S*. Tm and *K. oxytoca* CFUs after 24 h of co-cultivation with WT, tKO, sKO and cWT MK01 strains in cecal content media and (**i-j**) TLB (10 g/L). (**g-j**) The mean ± SEM of n = 3 independent biological experiments with n = 2-3 technical replicates are displayed. Source data

Toxin synthesis by various inhibitory and non-inhibitory KoSC strains was evaluated after culture in tryptone-lactose broth (TLB, 10 g l^−1^), a condition shown to induce toxin production^42^. Robust growth in this medium was verified for all strains (Extended Data Fig. 4a–c). Toxin was produced by all inhibitory isolates, and no production was detected in the tested panel of non-protective isolates (Fig. 3a–c and Extended Data Fig. 4d–f).

Next, we inactivated an essential *til* biosynthesis gene, *npsA*, in strain *K. oxytoca* MK01^27^. Specifically, we generated a total gene knockout (tKO) and a stop codon mutant (sKO). The sKO was utilized for the complementation of *npsA* (cWT). Analysis of culture supernatants (SNs) confirmed *npsA*-dependent toxin production for the MK01 strain (Fig. 3d,e). Ex vivo co-cultures in GF caecal-content media base (Fig. 3f and Extended Data Fig. 4g,h) and TLB (Fig. 3g and Extended Data Fig. 4i,j) showed that the mutant strains had a reduced ability to outcompete *S*. Typhimurium in both set-ups, while the WT and cWT strains inhibited *S*. Typhimurium (Fig. 3f,g).

**Extended Data Fig. 5:**
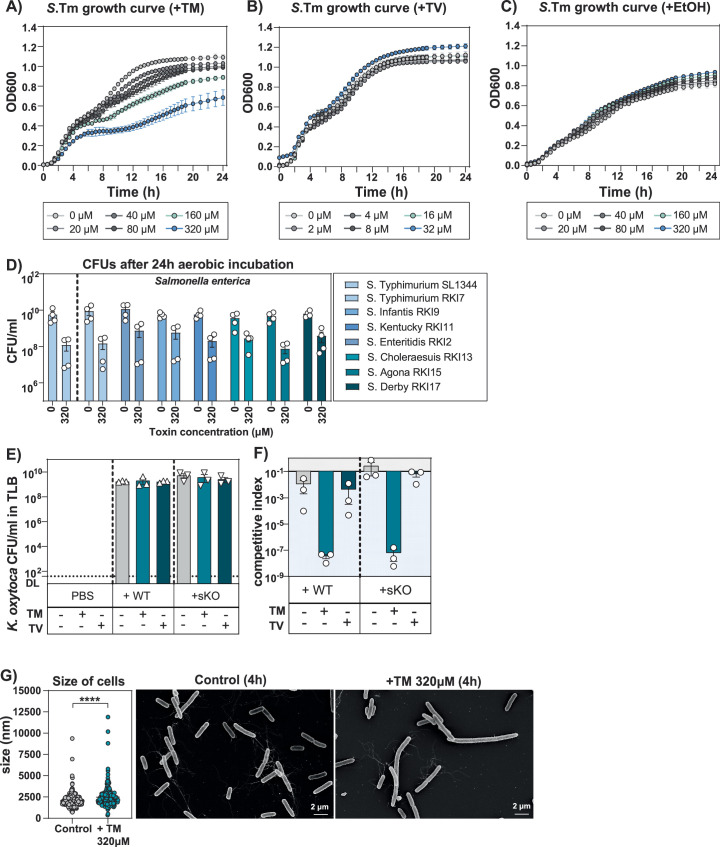
*K. oxytoca* toxins alone inhibit *S*. Tm partially, but complete inhibitory phenotype requires toxins and metabolic active bacteria. (**a**) OD_600_ of *S*. Tm in LB supplemented with various concentrations of TM (0 µM to 320 µM), (**b**) TV (0 µM to 32 µM) and (**c**) EtOH as a solvent control (0 µM to 320 µM) over 24 h. (**a-c**) The mean ±SEM of one out of two biological experiment with n = 2 technical replicates is displayed. (**d**) CFUs of various *Salmonella enterica* serovars in LB media supplemented with 320 µM TV or EtOH as solvent control. (**d**) The mean ± SEM of n = 2 independent biological experiments with n = 2-3 technical replicates per group are displayed. (E) *K. oxytoca* MK01 WT and sKO CFUs after co-cultivation with *S*. Tm in the absence or presence of 320 µM TM or 32 µM TV. (**f**) CI of *S*. Tm after 24 h of co-cultivation with *K. oxytoca* MK01 WT and sKO strains in TLB media supplemented with 320 µM TM or 32 µM TV. Solid line indicates starting ratio of bacteria (index = 0.1). (**e, f**) The mean ± SEM of n = 3 independent experiments with n = 2-3 technical replicates per group are displayed. (**g**) Representative scanning electron microscopy pictures at 4,000 X magnification of *S*. Tm in TLB media without and with 320 µM TM. Bar represents 2 µm. Average size of cells obtained from 10 microscopic images at randomly selected positions of *S*. Tm in TLB media without and with 320 µM TM after 4 h of aerobic cultivation. P-value indicated represents two-tailed Mann-Whitney U-test between groups with ****p < 0.0001. Source data

To investigate whether the toxins are sufficient to kill *S*. Typhimurium, we added chemically synthesized TM and TV to the growth media. The concentrations were chosen based on the quantitative data available for TM in mouse faeces^32,35^. Pronounced growth delay with the highest concentration of 320 µM TM and 10^1^- to 10^2^-fold reduced colony-forming units were observed in comparison to the control (Fig. 3h and Extended Data Fig. 5a–c). Consistent with previous observations for other bacteria, TM, but not TV, caused inhibition^32,35^; therefore, we focused mainly on TM in subsequent experiments. *Salmonella* strains from different serovars showed comparable susceptibility to TM (Extended Data Fig. 5d). It is interesting that the effect of TM was less pronounced compared to the bacterial co-culture assays (Extended Data Fig. 4i), suggesting that substrate competition or other interactions have an additive effect in the co-culture assays. To test this hypothesis, we co-cultivated *S*. Typhimurium with the toxin-deficient sKO strain and added TM or TV. Strikingly, if TM was added, the full inhibitory effect observed in WT co-cultures was retrieved also with the toxin-deficient sKO strain, highlighting that both the toxin and metabolic competition contributed to the full inhibitory effect in this set-up (Fig. 3i and Extended Data Fig. 5e,f). TM causes DNA breakage in eukaryotic cells and several bacterial species^32,34,35^. To assess how *S*. Typhimurium responds to TM, we first performed scanning electron microscopy of the bacteria observing a significant toxin-dependent elongation in a subset of *S*. Typhimurium cells (Extended Data Fig. 5g). Next, *S*. Typhimurium was exposed to the spent media SN of *K. oxytoca* MK01 WT and sKO strains followed by monitoring growth and survival using time-lapse microscopy of the bacteria in a microfluidic mother machine device (Fig. 3j–l). Following stable exponential growth during cultivation on TLB, we observed an immediate growth arrest of *S*. Typhimurium cells after exposure to SN (Fig. 3j,k). Upon switching back to fresh TLB after 4 h, *S*. Typhimurium cells exposed to sKO SN resumed growth immediately. On the contrary, the majority of *S*. Typhimurium cells exposed to WT SN were unable to re-grow, while other cells underwent lysis or filamentation (Fig. 3l and Supplementary Videos 1 and 2). These data show that toxin exposure inhibits cell division in *S*. Typhimurium^43^.

### Toxin production plays a major role in disturbed microbiota

**Extended Data Fig. 6:**
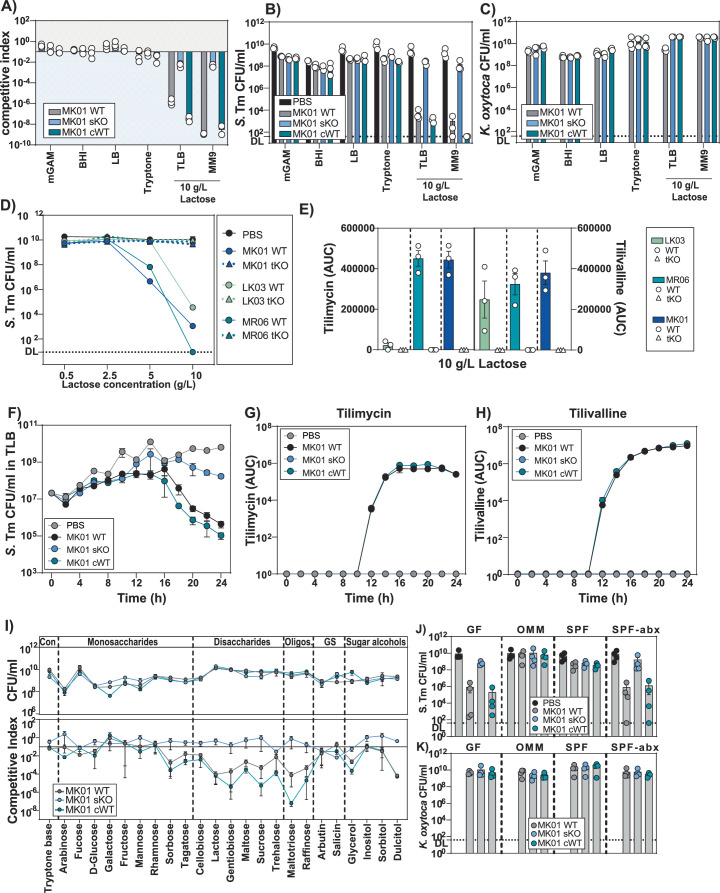
*K.oxytoca* toxin production plays a major role in disturbed microbiota settings with high amounts of free sugars. (**a**) CI of *S*. Tm after 24 h of co-cultivation with *K. oxytoca* MK01 WT, sKO and cWT strains in various semi-defined and defined culture media. (**b**) Corresponding *S*. Tm and (**c**) *K. oxytoca* CFUs after co-cultivation in various semi-defined and defined culture media. (**a-c**) The mean ± SEM of n = 3 independent experiments with n = 2-technical replicates per group are displayed. (**d**) *S*. Tm was co-cultured with three different *K. oxytoca* WT and tKO mutant strains (MK01, LK03 and MR06) in TLB with various concentrations of lactose. Resulting *S*. Tm CFUs are displayed. The mean ± SEM of n = 2 independent experiments with n = 2-technical replicates per group are displayed. (**e**) Area under curve (AUC) for TM and TV in sample supernatants of different WT and tKO strains of *K. oxytoca* after 24 h of co-cultivation with *S*. Tm in TLB medium with 10 g/l lactose. The mean ± SEM of three independent cultures are displayed. (**f**) *S*. Tm CFUs after various time points (every 2 h over 24 h) of co-cultures with MK01 WT, sKO and cWT strains in TLB and (**g**) corresponding AUCs for TM and (**h**) TV (F-H) The mean ± SEM of n = 2 independent experiments with n = 2 technical replicates are displayed. (**i**) *K. oxytoca* CFUs (upper panel) and corresponding CI (lower panel) of co-cultures with *S*. Tm in tryptone broth with various mono-, di- and oligo-saccharides as carbon sources (10 g/L). Solid line indicates starting ratio of bacteria (index = 0.1). The mean ± SEM of n = 2-3 independent experiments performed in n = 3 technical replicates are displayed. (**j**) *S*. Tm and (**k**) *K. oxytoca* CFUs after co-cultivation in sterile filtered cecal content supernatants of GF, OMM^12^, SPF and antibiotic treated SPF mice (abx). Dashed lines display limit of detection. (**j-k**) The mean ± SEM of four independent experiments with n = 2-technical replicates per group are displayed. Source data

Media containing soy or high concentrations of lactose or glucose were previously used to induce toxin production in vitro in diverse KoSC strains^42,44,45^. To better characterize the nutrient environment promoting toxin production in *K. oxytoca*, we utilized growth inhibition of *S*. Typhimurium as an indirect read-out. Strong inhibition of *S*. Typhimurium (>10^5^-fold reduction) was detected only in complex or minimal media with high lactose concentrations, but not in any of the other rich and complex media (<10^2^-fold reduction) (Extended Data Fig. 6a–c). Next, we tested three different *K. oxytoca* WT and *ΔnpsA* tKO strains (MK01, LK03 and MR06) in tryptone media with different lactose concentrations. A toxin-dependent inhibition was observed for all strains at 10 g l^−1^ and two of three strains (MR06, MK01) at 5 g l^−1^ showing strain-to-strain variability (Extended Data Fig. 6d). Semi-quantitative analyses of toxin production showed lower production of TM and TV by *K. oxytoca* LK03 compared to MR06 and MK01 (1.3-fold to 1.5-fold lower) (Extended Data Fig. 6e). We next monitored *S*. Typhimurium counts and toxins over time (Extended Data Fig. 6f–h). Toxin production started after 12 h of co-cultivation and accumulated over time, which was followed by a strong toxin-dependent reduction of *S*. Typhimurium starting at 14 h and peaking at the end of the experiment (Extended Data Fig. 6f–h).

**Fig. 4:**
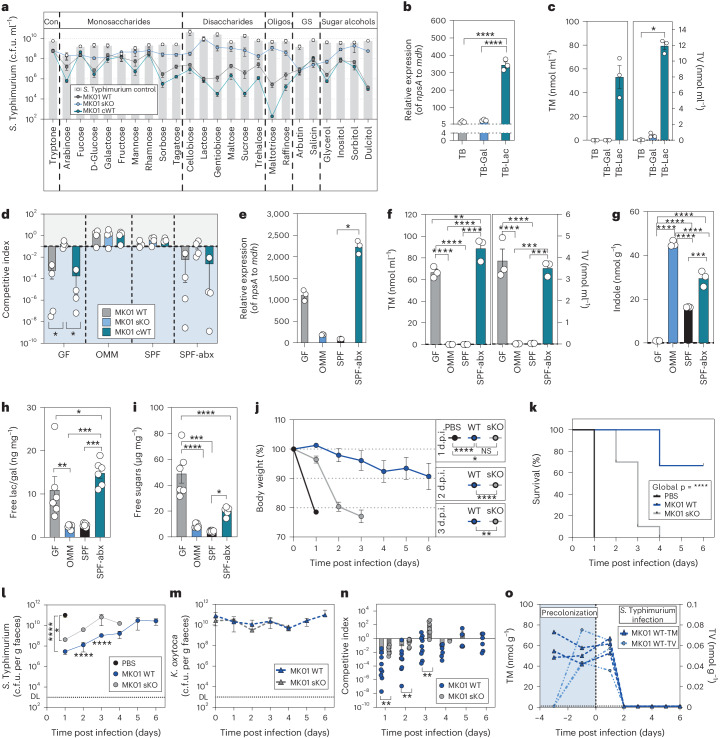
*K. oxytoca* toxin production is regulated through sugar availability and is important in GF mice. **a**, Colony-forming units of *S*. Typhimurium grown alone or in co-culture with MK01 WT, sKO or cWT in tryptone with various sugars. Mean and s.e.m. of *n* = 3 experiments with *n* = 3 replicates. Con, control; GS, glycosides. **b**, Relative gene expression of *npsA* in MK01 WT in TB with added galactose or lactose. Mean ± s.e.m. one experiment with *n* = 3 samples with *n* = 3 replicates. Gal, galactose; Lac, lactose. **c**, Quantification of TM and TV in the SN of MK01 WT in TB media with galactose or lactose. Mean ± s.e.m. of *n* = 3 independent cultures. **d**, CI of *S*. Typhimurium in co-cultures with *K. oxytoca* MK01 WT, sKO or cWT in various sterile caecal contents. Mean ± s.e.m. of *n* = 4 experiments with *n* = 2 replicates. The dashed line indicates the inoculation ratio. **e**, Relative gene expression of *npsA* in MK01 WT in various caecal contents. Mean ± s.e.m. of one representative experiment from *n* = 3 samples with *n* = 3 replicates. **f**,**g**, Quantification of TM and TV (**f**) and of indole (**g**) in the SN of MK01 WT in various caecal contents. Mean ± s.e.m. of *n* = 3 independent cultures. **h**,**i**, Free lactose/galactose levels (**h**) and total free carbohydrate levels (**i**) from SNs of caecal content. Mean ± s.e.m. of *n* = 6 mice measured in duplicates. **j–o**, GF mice were colonized with MK01 WT or sKO or left untreated 4 days before infection. On day 0 mice were orally infected with *S*. Typhimurium, and BWL, survival and faecal colonization were monitored until day 6 p.i. **j**, BWL over the time of infection. d.p.i., days post infection; NS, not significant. **k**, Survival of various *S*. Typhimurium infected groups. Log-rank (Mantel–Cox) test between curves with ****P < 0.0001. **l**–**n**, Faecal colony-forming units of *S*. Typhimurium (**l**) and *K. oxytoca* (**m**), and resulting CI at various time points (**n**). DL, level of detection. The solid line indicates equal amounts of bacteria (index = 1). **o**, Faecal TM and TV in WT colonized animals. In **j**–**n**, mean ± s.e.m. of *n* = 2 experiments with *n* = 8 (PBS), *n* = 9 (WT) or *n* = 10 (sKO) mice per group. In **b** and **e**, ordinary one-way ANOVA with Holm–Šídák’s multiple comparisons test with *P < 0.05, ****P < 0.0001. In **d** and **f**–**i**, ordinary one-way ANOVA with Tukey’s multiple comparisons test between groups with *P < 0.05, **P < 0.01, ***P < 0.001 and ****P < 0.0001. In **c** and **j**–**n**, one-way ANOVA with Dunn’s multiple comparisons test between groups. In **j** and **l**–**n**, two-tailed Mann–Whitney *U*-test between two groups with *P < 0.05, **P < 0.01, ***P < 0.001 and ****P < 0.0001. See also Extended Data Fig. 6. Source data

To investigate whether only lactose or also other simple sugars induce toxin-dependent inhibition, we screened a panel of sugars for their toxin-inducing capacities (Fig. 4a and Extended Data Fig. 6i). Toxin-dependent inhibition of *S*. Typhimurium was observed for various simple sugars and was most pronounced in sorbose, lactose, gentiobiose, sucrose, trehalose, maltotriose, raffinose and dulcitol (reduction from 10^3^-fold to 10^6^-fold). Notably, toxin-independent inhibition of *S*. Typhimurium, that is, equal CI for the WT, cWT and sKO strains of MK01, was observed for various sugars including the glucosides arbutin and salicin, and galactose. This indicates that toxin production could be induced by various but not all simple carbohydrates frequently found in the human and mouse gut. Moreover, the 10-fold to 100-fold reduction of *S*. Typhimurium colony-forming units in co-culture with the sKO strain observed with several sugars corroborates that substrate competition plays a role in vitro in addition to the toxin (Fig. 4a and Extended Data Fig. 6i). Next, we quantified *npsA* gene expression after 8 h of growth and the total amount of TM and TV after 24 h. The gene expression of *npsA* increased significantly with lactose compared to galactose or tryptone broth (TB) alone (Fig. 4b). Correspondingly, lactose-containing TB showed a three-fold increase in *til* metabolites after 24 h compared to galactose-containing TB (Fig. 4c), while no *til* metabolites were detected in TB alone. Together, these results show that a variety of sugars found in the gut can induce toxin production in a concentration-dependent manner in vitro, with some sugars such as lactose being more effective than others.

### The contribution of toxin production is microbiota-dependent

To analyse in vitro whether toxin-inducing conditions are present in different ecological contexts, we utilized the ex vivo assay using caecal contents from mouse lines with varying microbiome compositions. Strikingly, we observed inhibition of *S*. Typhimurium in SPF-abx and GF caecal contents, but not in the OMM^12^ or antibiotic naive SPF contents (Fig. 4d) despite similar *K. oxytoca* growth in all settings (Extended Data Fig. 6j,k). Quantification of *npsA* transcripts and TM and TV production revealed that the caecal content of GF and SPF-abx mice support a strong increase of *npsA* gene expression (Fig. 4e) and production of TM and TV relative to OMM^12^ or SPF mice (Fig. 4f). While specific sugars induce toxin production, microbiota-derived indole has been shown to repress toxin production^44^. Indole was detectable in all conditions except in GF content (Fig. 4g), yet indole levels did not correlate with toxin production. In turn, quantification of free lactose/galactose and total carbohydrates using enzyme-linked immunosorbent assay kits revealed 4-fold to 6-fold higher lactose/galactose concentrations in the caecal content of GF and SPF-abx compared to OMM^12^ and SPF mice (Fig. 4h). Similarly, total carbohydrate concentrations were highest in GF (5-fold to 12-fold) and SPF-abx mice (2-fold to 4.5-fold) compared to OMM and SPF mice (Fig. 4i). Thus, compared with indole levels, sugar availability better correlates with toxin production in these models.

To assess how much of the protective phenotype observed in vivo is due to toxin production, we utilized GF mice, which offer the highest carbohydrate availability. Mice were precolonized, either with the *K. oxytoca* WT or the sKO strain for 4 days or left untreated before all animals were infected with *S*. Typhimurium. We observed attenuated BWL (Fig. 4j) of GF mice in both *K. oxytoca*-treated groups with significantly longer survival in the WT group (mean survival = 6 days) compared to the sKO group (mean survival = 3.5 days) (Fig. 4k). Similarly, the expansion of *S*. Typhimurium was slower in both *K. oxytoca* colonized groups but more strongly inhibited in the WT group (Fig. 4l) despite equal colonization of *K. oxytoca* throughout the experiment (Fig. 4m). This led to more effective inhibition of *S*. Typhimurium by WT compared to the sKO strain (Fig. 4n). Moreover, TM and TV were detectable throughout the experiment until day 2 p.i. (Fig. 4o). Taken together, in the absence of a microbiota, *K. oxytoca* inhibits *S*. Typhimurium in a largely toxin-dependent manner.

**Fig. 5:**
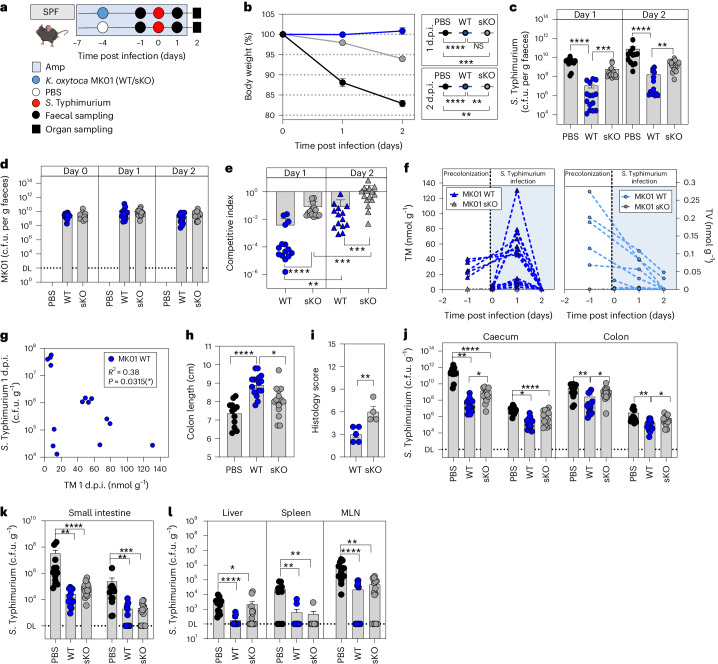
*K. oxytoca* toxin production plays an intermediate role in disturbed but complex microbiota settings. **a**, Ampicillin-treated SPF mice were colonized with *K. oxytoca* MK01 WT, sKO or left untreated 4 days before infection. On day 0, mice were orally infected with *S*. Typhimurium, and BWL and faecal colonization were monitored until day 2 p.i. Organs were sampled for examination of *S*. Typhimurium burden on day 2 p.i. Amp, ampicillin. **b**, Resulting BWL on day 1 and day 2 p.i. **c**, *S*. Typhimurium burden in the faeces on day 1 and day 2 p.i. d.p.i., days post infection. **d**, Corresponding levels of *K. oxytoca* MK01 WT or sKO in the faeces of the mice. DL, level of detection. **e**, Resulting CI of *K. oxytoca* MK01 WT and sKO colonized animals. *P* values indicated represent two-tailed Mann*–*Whitney *U*-test between WT and sKO or Wilcoxon matched-pairs signed rank test within each group comparing the different time points. **f**, Absolute quantification of faecal TM and TV at various time points of the experiment in MK01 WT and sKO colonized SPF-abx animals. **g**, Correlation of faecal TM levels and *S*. Typhimurium burden in the faeces on day 1 p.i. of *K. oxytoca* MK01 WT colonized mice. Pearson’s correlation coefficient *R*^2^ = 0.3088 and *P* = 0.0315 (two-tailed test). **h**, Colon length on day 2 p.i. **i**, Histological inflammation score of the proximal colon. Mean ± s.e.m. of one experiment with *n* = 4 (PBS) or *n* = 5 (WT/sKO) mice per group. **j**–**l**, Resulting *S*. Typhimurium burden in the lumen and tissue of the caecum and colon (**j**), the small intestine (**k**) and extra-intestinal organs including liver, spleen and mesenteric lymph nodes (MLN) (**l**). In **b**–**h** and **j**–**l**, mean ± s.e.m. of *n* = 3 experiments with *n* = 12 (PBS), *n* = 15 (WT/sKO) mice per group are displayed. *P* values indicated represent ordinary one-way ANOVA with Dunn’s multiple comparisons test (**b**, **c**, **h** and **j**–**l**) or two-tailed Mann–Whitney *U*-test (**e** and **i**) with *P < 0.05, **P < 0.01, ***P < 0.001 and ****P < 0.0001. Source data

We next assessed the CR phenotype in mice following antibiotic disruption of the gut microbiome. SPF-abx mice were precolonized with WT or sKO strains and then challenged with *S*. Typhimurium (Fig. 5a). We observed a less pronounced but significant difference between WT and sKO colonized animals regarding BWL (Fig. 5b) and faecal *S*. Typhimurium levels (Fig. 5c), despite equal *K. oxytoca* levels (Fig. 5d), resulting in more effective inhibition by the WT strain compared to sKO (Fig. 5e). Notably, inhibition with each strain dropped significantly from day 1 to day 2 p.i. parallel to loss of faecal TM and TV (Fig. 5f). It is interesting that we observed highly variable toxin concentrations on day 1 p.i. in the faeces of the different animals, which seem to correlate with the observed level of inhibition of *S*. Typhimurium on this day (Fig. 5g) giving a possible explanation for the in-group variability observed in the *K. oxytoca* WT colonized mice. Thus, while toxin production in the gut is maintained over a short period in this model, a significant difference between WT and sKO strains was detected concerning colon shortening, colonic inflammation scores (Fig. 5h,i) and expansion of *S*. Typhimurium in gastrointestinal and extra-intestinal organs (Fig. 5j,k). Nonetheless, the sKO strain mediated a significant reduction of *S*. Typhimurium in most organs and faeces. In summary, the experiments in GF and SPF-abx mice showed that toxin production plays an important but non-exclusive role in CR in vivo.

### Toxin production is dispensable in complex communities

**Fig. 6:**
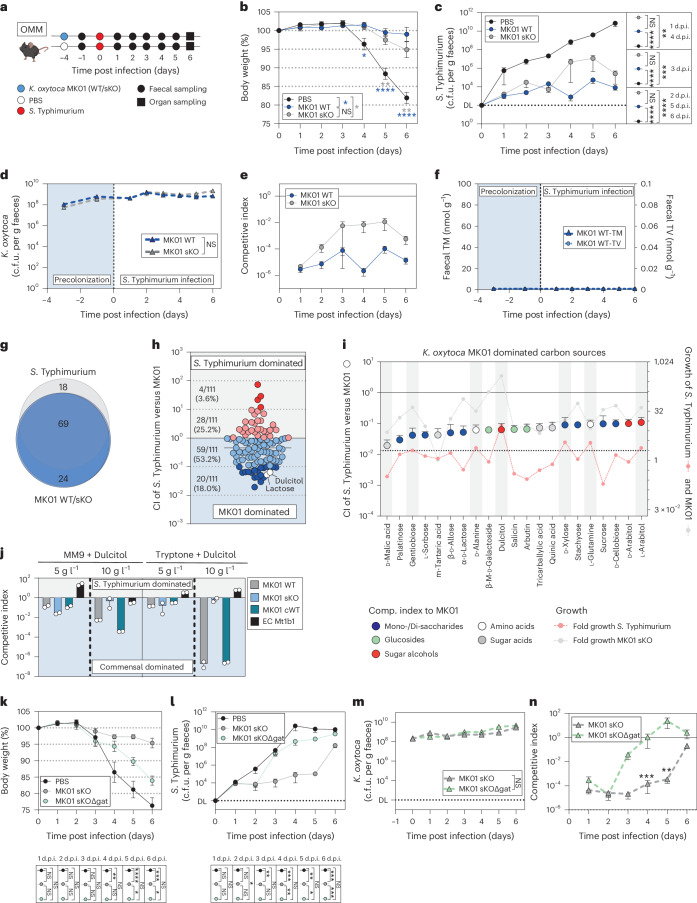
Protective phenotype of *K. oxytoca* in homeostatic microbiota is dependent on carbohydrate competition. **a**, OMM^12^ mice were colonized with *K. oxytoca* MK01 WT, sKO or left untreated 4 days before infection. On day 0, mice were orally infected with *S*. Typhimurium, and BWL, faecal colonization and microbiome composition were monitored until day 6 p.i. when organs were sampled. **b**–**e**, BWL (**b**), faecal colony-forming units of *S*. Typhimurium (**c**) and *K. oxytoca* (**d**) and CI of WT and sKO colonized mice (**e**). DL, level of detection; d.p.i., days post infection. **f**, Faecal TM and TV over time. (**b–f**) Mean ± s.e.m. of *n* = 3 experiments with *n* = 12 (PBS/sKO) or *n* = 13 (WT) mice per group. **g**, Venn diagram displaying carbon source overlap of *S*. Typhimurium and MK01 WT/sKO based on *n* = 4 independent measurements. **h**, CI of *S*. Typhimurium after co-cultivation with sKO in 111 carbon sources in a 1:1 ratio. **i**, The top 20 *K. oxytoca*-dominated carbon sources displayed as CI (left *y*-axis) in comparison to the growth of both bacteria in single cultures (right *y*-axis). The dashed line indicates active growth >2. In **h** and **i**, mean ± s.e.m. of *n* = 4 independent measurements. **j**, CI of *S*. Typhimurium co-cultures with MK01 WT, sKO, cWT, and *E. coli* Mt1b1 strains in MM9 or TB supplemented with dulcitol. Mean ± s.e.m. of *n* = 2 experiments with *n* = 2 technical replicates are displayed. **k**–**n**, OMM^12^ mice were colonized with *K. oxytoca* MK01 sKO, sKO*ΔgatABC* or left untreated 4 days before infection. On day 0, mice were orally infected with *S*. Typhimurium, and BWL and faecal colonization was monitored until day 6 p.i. when organs were sampled. BWL (**k**); faecal colony-forming units of *S*. Typhimurium (**l**) and *K. oxytoca* (**m**); and resulting CI of *K. oxytoca* colonized mice (**n**). The mean and s.e.m. of *n* = 2 experiments with *n* = 7 (PBS) or *n* = 8 (sKO/ sKO*ΔgatABC*) mice per group. In **b**, **c**, **k** and **l**, ordinary one-way ANOVA with Dunn’s multiple comparisons test between groups with *P < 0.05, **P < 0.01, ***P < 0.001 and ****P < 0.0001. In **d**–**f**, **m** and **n**, two-tailed Mann*–*Whitney *U*-test with *P < 0.05, **P < 0.01, ***P < 0.001 and ****P < 0.0001. In **h**, **i**, **j** and **n**, the solid line indicates the starting ratio of bacteria (index = 1). See also Extended Data Figs. 7–10. Source data

**Extended Data Fig. 7:**
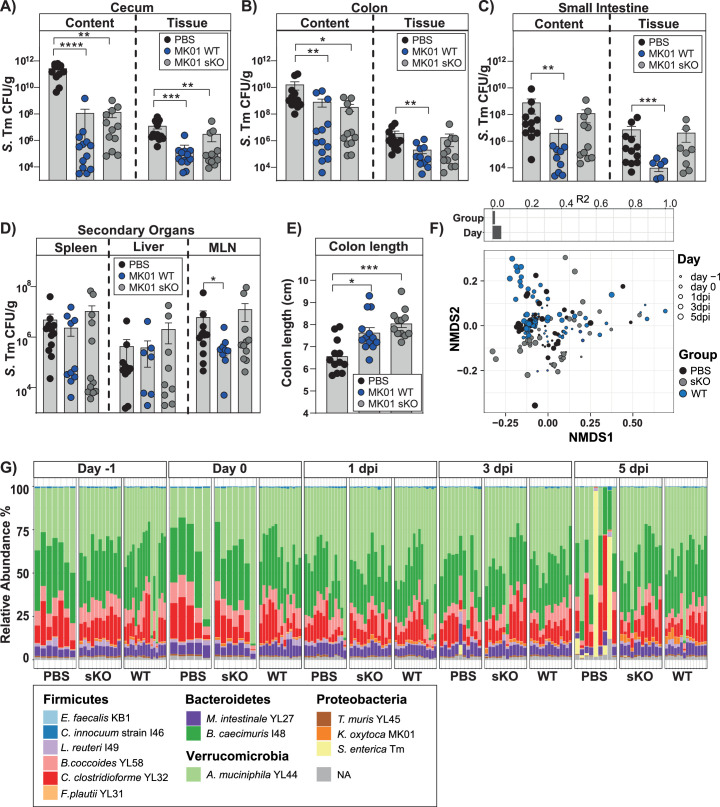
Protective phenotype in OMM^12^ mice is largely toxin-independent. Mice were precolonized with WT MK01 or sKO or left untreated before infection with *S*. Tm. *S*. Tm burden in the lumen and tissue of (**a**) cecum, (**b**) colon, (**c**) small intestine and (**d**) various secondary organs including spleen, liver and MLN of infected OMM^12^ mice at day 6 p.i. (**e**) Colon length at day 6 p.i. in *K. oxytoca* colonized mice and control mice. (A-E) The mean ± SEM of n = 3 independent experiments with n = 12 (PBS/sKO) and n = 13 (WT) mice per group. P values indicated represent Ordinary One-Way ANOVA with Dunn’s multiple comparisons test with *p < 0.05, **p < 0.01, ***p < 0.001 and ****p < 0.0001. (**f**) β-diversity of OMM^12^ mice using non-metric multidimensional scaling (NMDS). Different groups and sampling time points are indicated. Individual effect size of tested covariates is indicated. To calculate the variance explained by individual factors such as group and time point, a permutational multivariate analysis of variance (ADONIS) was used. A significant effect was dedicated when *p* < 0.05 and *R*^2^ > 0.10 (equivalent to 10 % of explained variance) was observed. (**g**) Microbiome composition (relative abundance) of each single mouse in different groups (PBS, WT and sKO-colonized) at various time points of the experiment are displayed on species level. (**f-g**) Data derived from n = 3 independent experiments with n = 14 (PBS), n = 12 sKO, n = 16 (WT) mice. Source data

While *K. oxytoca* provided CR against *Salmonella* in OMM^12^ mice, caecal content from the mice failed to induce toxin production. To test whether toxin production contributes in a homeostatic, non-antibiotic-disturbed gut community, OMM^12^ mice were precolonized with either the WT or sKO strains or left untreated before all animals were infected with *S*. Typhimurium (Fig. 6a). In this model, only minor differences were observed between both strains regarding BWL (Fig. 6b), faecal *S*. Typhimurium colonization (Fig. 6c), tissue infiltration and inflammation (Extended Data Fig. 7a–e). As observed in GF and SPF-abx mice, *K. oxytoca* colonization levels were independent of toxin production (Fig. 6d). Nevertheless, the WT strain tends to protect better in comparison to the sKO strain (Fig. 6e) even though potentially due to 10-fold to 100-fold lower population densities of *K. oxytoca* in this model, *til* metabolites were below the level of quantitation (Fig. 6f). While these results do not show that the toxins are not produced in OMM^12^ mice, they suggest that the contribution of toxin production to microbial competition is microbiota-dependent and less pronounced than in GF and SPF-abx mice.

**Extended Data Fig. 8:**
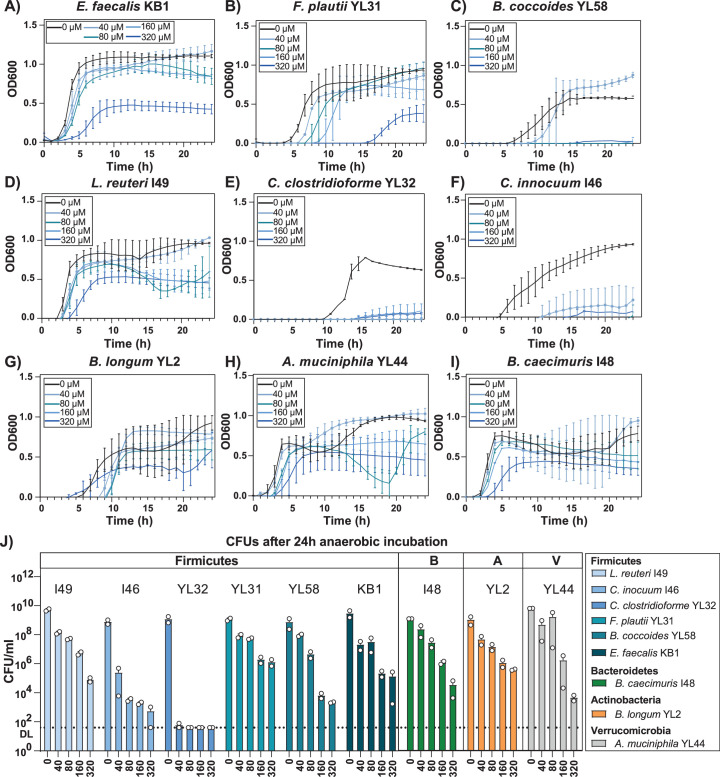
TM has broad spectrum activity against gut members of the OMM^12^ microbiome *in vitro.* (**a-i**) Growth curves of various gut bacteria of the OMM^12^ community in presence of various TM concentrations ranging from 0 µM to 320 µM. Results represent the mean ± SEM of two (40 mM – 320 mM) or n = 3-4 (0 mM) independent measurements performed in n = 2 technical replicates replicates. (**j**) Corresponding CFUs of the same bacteria cultivated for 24 h under anaerobic conditions in BHI medium supplemented with TM concentrations ranging from 0 µM to 320 µM. Bacteria are sorted based on their phylum. Results represent the mean ± SEM of n = 2 biological experiments performed in n = 2 technical replicates. Source data

As TM inhibits diverse commensal bacteria^35^, we next analysed microbiota composition during infection but failed to detect any significant differences between the WT and sKO colonized mice. Yet, a significant shift in the community in the infected control mice due to the pronounced inflammation and *S*. Typhimurium expansion was detected (Extended Data Fig. 7f,g). To exclude that OMM^12^ members are insensitive to TM, representative members were cultured in the presence of 40–320 µM TM. Growth of all tested members was reduced surpassing even the TM impact on *Salmonella* (Extended Data Fig. 8a–j). Together these results show that toxin production is not essential, and in a system of more complex microbial interactions additional mechanisms promote resistance, including most likely nutrient utilization.

### CR relies on nutrient competition in homeostatic communities

**Extended Data Fig. 9:**
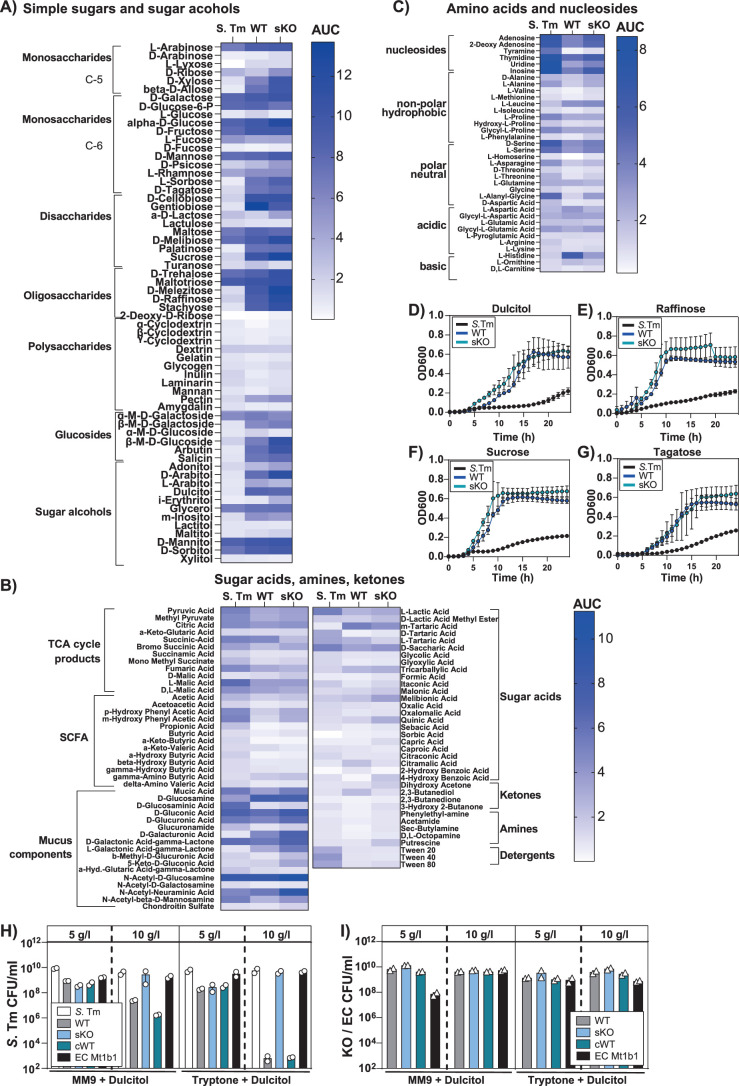
*K. oxytoca* and *Salmonella* have distinct but partially overlapping carbohydrate preferences and likely compete for various substrates. (**a-c**) Heatmaps displaying growth displayed as area under curve in selected carbon sources for *S*. Tm in comparison with *K. oxytoca* MK01 WT and the sKO mutant after 24 h aerobic incubation using the Biolog microarrays PM1 and PM2a. Sugars are grouped based on their chemical structure with simple sugars and sugar alcohols displayed in (**a**), sugar acids, amines and ketones displayed in (**b**) and amino acids and nucleosides displayed in (**c**). Color scale bar shows AUC over the period of 24 h aerobic incubation. (**a-c**) Results represent mean (average growth) values of n = 3 independent measurements. (**d-g**) Growth curves of *S*. Tm and MK01 WT and sKO in selected carbon sources including (**d**) dulcitol, (**e**) raffinose, (**f**) sucrose and (**g**) tagatose. Results represent mean ± SEM growth values of n = 3 independent measurements with n = 2 technical replicates. (**h**) *S*. Tm CFUs and (**i**) *K. oxytoca / E. coli* CFUs in MM9 medium supplemented with 5 g/l or 10 g/l dulcitol after 24 h of co-cultivation in a 1:1 ratio. Results represent mean ± SEM of n = 2 independent experiments performed in n = 2 technical replicates. Source data

**Extended Data Fig. 10:**
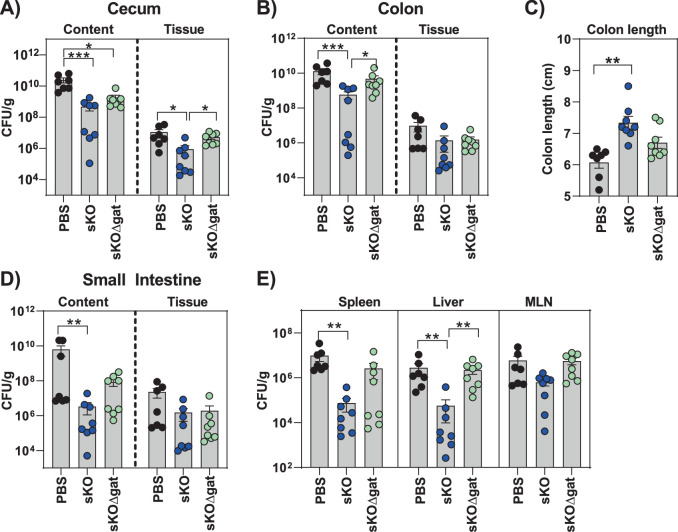
Metabolic competition for dulcitol is a key factor for protection against *S*. Tm. OMM^12^ mice were colonized with 5 × 10^8^ CFUs of *K. oxytoca* MK01 sKO, sKO*ΔgatABC* or left untreated four days before infection. At day 0 mice were orally infected with 5×10^5^ CFUs of *S*. Tm and BWL and fecal colonization were monitored until day 6 p.i. Organs were sampled at day 6 p.i. (**a**) Resulting CFUs in the lumen and tissue of cecum and (**b**) colon. (**c**) Colon length at day 6 p.i. and (**d**) resulting CFUs in the lumen and tissue of the small intestine and (**e**) extra-intestinal organs including spleen, liver and MLN. (**a-e**) The mean and SEM of two independent experiments with n = 7 (PBS) or n = 8 (sKo/WT) mice per group are displayed. P values indicated represent Ordinary One-Way ANOVA with Dunn’s multiple comparisons test with *p < 0.05, **p < 0.01, ***p < 0.001 and ****p < 0.0001. Source data

We next used the phenotypic Biolog microarray to screen for carbohydrates that *K. oxytoca* utilizes to compete against *S*. Typhimurium. *S*. Typhimurium, *K. oxytoca* MK01 WT and sKO grew in 89, 97 and 93 carbon sources, respectively. Overall, the utilization of 69 carbon sources was shared by all strains, while 18 others could be utilized by *S*. Typhimurium alone and 24 solely by *K. oxytoca* MK01 WT and sKO (Fig. 6g). *K. oxytoca* preferred simple sugars and sugar alcohols (Extended Data Fig. 9a–c), while *S*. Typhimurium preferred sugar acids, mucus components, amino acids and nucleosides. Moreover, various simple sugars frequently found in the mouse gut, for example, dulcitol, raffinose, sucrose and tagatose, support the comparatively rapid expansion of MK01 (Extended Data Fig. 9d–g). MK01 thus appears able to use more carbon sources and some more effectively. To test this hypothesis, *S*. Typhimurium and MK01 sKO were co-inoculated in a 1:1 starting ratio in Biolog microarray plates. We specifically utilized the sKO strain to focus on toxin-independent metabolic competition between both strains. The majority (79/111 = 71.2%) of the tested carbon sources allowed *K. oxytoca* MK01 to outcompete *S*. Typhimurium (Fig. 6h). From those, 21 supported >10-fold higher ratios of MK01 to *S*. Typhimurium (Fig. 6i). Most of those carbon sources were exclusively utilized by *K. oxytoca*; however, five could be utilized by both bacteria of which dulcitol showed the strongest growth for *S*. Typhimurium when grown individually (faint red dots with dashed line). Dulcitol utilization by an *E. coli* strain was previously shown to provide CR against *S*. Typhimurium in the OMM^12^ microbiome^41^. Co-cultivation of *S*. Typhimurium with *K. oxytoca* MK01 strains in medium revealed a toxin-independent 10^1^-fold to 10^2^-fold greater inhibition by *K. oxytoca* at low dulcitol concentrations (5 g l^−1^) and an even more pronounced, toxin-dependent effect at higher concentrations (10 g l^−1^) (Fig. 6j and Extended Data Fig. 9h,i). Notably, the *E. coli* Mt1b1 strain was less competitive against *S*. Typhimurium than *K. oxytoca* MK01 at all tested conditions, even at low dulcitol concentration, indicating more efficient dulcitol utilization by MK01 compared to *S*. Typhimurium and *E. coli* Mt1b1 (Fig. 6j). Finally, to investigate whether dulcitol utilization is a critical factor for toxin-independent protection against *S*. Typhimurium in OMM^12^ mice, we generated a dulcitol utilization-deficient strain in the MK01 sKO background to exclude any potential influence of the toxin. Mice were colonized with the sKO and sKOΔ*gatABC* strains as previously described. Indeed, the inability to utilize dulcitol caused loss of the resistance phenotype in the mutant as measured by a significantly elevated BWL (Fig. 6k) and increased *S*. Typhimurium colonization in the faeces (Fig. 6l) despite equal colonization of both strains (Fig. 6m) leading to a diminished CI (Fig. 6n) and reduced colony-forming units in gastrointestinal organs (Extended Data Fig. 10a–e).

Taken together, these results show that, depending on the microbial context and the quantity of available sugars, *K. oxytoca* protection against *S*. Typhimurium relies on a variable combination of toxin dependence and substrate-driven competition.

## Discussion

It is now well recognized that members of the KoSC are frequent colonizers of the infant gut and are also present in adults^23,24^, yet, their contributions to the developing microbiome and host health remain largely unknown. In this Article, we describe two independent but interconnected mechanisms of toxin-dominated and metabolic-dominated competition between commensal KoSC strains and *S*. Typhimurium in mouse models representing different ecological states. While OMM^12^ mice represent a stable, low-complexity community^46^ with low levels of free total carbohydrates and no detectable TM in the faeces, the antibiotically disturbed gut environment of SPF mice and the gut environment in GF mice have increased levels of available carbon sources, detectable amounts of TM in the faeces and 100-fold higher population densities of *K. oxytoca* in vivo. Despite these differences in population size, *K. oxytoca* provided CR against *S*. Typhimurium in all three models. In GF and antibiotic-treated SPF mice, toxin production presented a decisive advantage for the host, enabling survival to an otherwise lethal challenge with *S. enterica* in vivo. This ‘beneficial’ effect of the toxin adds another dimension to the functional diversity of natural compounds with detrimental effects for the host on the one side^22,24,30–32^ and potent antimicrobial activity against major human pathogens on the other side.

Infections with *S. enterica* serovars present a major health threat, especially in the infant population^37,38^. Here we show that many human *K. oxytoca* and *K. grimontii* isolates restrict the growth of various clinical *S. enterica* strains. Specifically, 78 of 81 toxin-producing strains, frequently isolated from the gut of healthy preterm infants and toddlers, inhibited *S*. Typhimurium ex vivo, while none of 47 non-toxinogenic KoSC strains could inhibit *S*. Typhimurium to similar levels. The observation that TM shows antibacterial activity across several bacterial phyla^35^ and that toxin proficiency is widespread in strains isolated from individuals with no apparent enteric disease supports the premise that the *til* locus is primarily maintained for interbacterial competition. Similarly, colibactin, a genotoxin produced by *E. coli* strains including the probiotic *E. coli* Nissle, has detrimental effects on host cells but also provides antibacterial activity against pathogens such as *S*. Typhimurium, *C. rodentium*, or *Vibrio cholerae*^9,36^. Thus, TM and colibactin share biological roles with other antimicrobial proteins and effector molecules that can target multiple species and thereby increase competitiveness in the gut^16,47–49^.

Independent of toxin production, the contribution of *K. oxytoca* to the gut ecosystem is illustrated by the protection it confers against *S*. Typhimurium in gnotobiotic mice harbouring commensal bacteria with complementary metabolic functions^50^. In this OMM^12^ model, *K. oxytoca*-mediated protection was largely toxin independent and arises from the metabolic potential of *K. oxytoca*, which is comparably higher than that of *S*. Typhimurium. Competition for several different substrates favoured *K. oxytoca* MK01 compared to *S*. Typhimurium in vitro, but dulcitol played a particular role in vivo.

Importantly, as the ‘nutrient–niche hypothesis’ was postulated first^51,52^, numerous studies have underscored the relevance of metabolism-related mechanisms mediated by microbe–microbe interaction for effective colonization resistance. In particular, the importance of low abundant commensal keystone species in the competition for substrates with medically relevant pathogens has been studied. Examples include *K. oxytoca* (strain MK01) protecting against *K. pneumoniae*^4^, and *Klebsiella* sp. AR0112 protecting against *E. coli* and *Salmonella*^18^. Moreover, cooperative nutrient competition resulting in the limitation of key carbon sources and electron acceptors through the interaction of enterobacteria and other commensal bacteria was found to contribute to CR^4^^,41,53^. These examples are consistent with the so-called ‘restaurant hypothesis’, which describes available nutrients in the gut as important regulators of initial perinatal microbial colonization, particularly *E. coli*^54–56^. These initial colonizers comprising facultative anaerobic bacteria, such as *E. coli* and *Klebsiella* spp., play an important role in the neonatal gut while representing minor components in the healthy adult gut ecosystem^57–59^. Due to their facultative anaerobic nature and remarkable metabolic flexibility, the presence or absence of enterobacterial keystone species is expected to confer functional relevant variability in the developing or disturbed human gut microbiome. This includes community-dependent consumption of oxygen and carbohydrates, alteration of the pH, redox potential and production of carbon dioxide, nutrients and inhibitory compounds ultimately making the gut habitat more or less suitable for colonization by strict anaerobes^54,55^. These in turn degrade complex carbohydrates making them accessible for enterobacterial consumption. Here we demonstrate that the environment-dependent production of antibacterial secondary metabolites is intimately linked to the availability of carbon sources and further show the potential relevance of these to interspecies interactions.

Microbial competition is relevant not only for the interaction between *S*. Typhimurium and *K. oxytoca* but also for the interaction between *K. oxytoca* and the surrounding microbial ecosystem. Members of the OMM^12^ community seem to be able to restrict available carbon sources to levels preventing the production of detectable amounts of TM which would otherwise strongly inhibit these gut members. While this is by itself an interesting biological observation, it might also be a relevant finding for future intervention strategies to prevent the negative effects of acute or chronic toxin production.

## Methods

### Reagent and resource sharing

Further information on resources and reagents are available from the corresponding author on request.

### Experimental model and subject details

#### Human data collection

Human sample and data collections for healthy participants have been performed in agreement with the guidelines of the Helmholtz Center for Infection Research, Braunschweig, Germany, the Ethics Committee Lower Saxony (permit number 8629_BO_K_2019; number 8750_BO_K_2019) and the European Data Protection Laws (Europäische Datenschutz-Grundverordnung DSGVO). Patients or patient characteristics (sex, gender, ethnicity) were not part of the analysis, and no patient data are reported. We analysed bacterial isolates, which were selected based on the following criteria: strain, antibiotic resistance, isolation from patients and no environmental bacteria. Bacterial isolates fulfilling the relevant criteria were stored at the Institute of Medical Microbiology and Hospital Epidemiology. Pseudonymized bacterial strains were used for analysis according to the ethics vote covering this study: 10392_BO_K_2022. All human donors have signed a letter of informed consent in accordance with the World Medical Association Declaration of Helsinki (version 2013). Metadata available for all isolates derived from human donors are listed in Supplementary Table 1.

#### Mice

All animal experiments have been performed in agreement with the guidelines of the Helmholtz Center for Infection Research, Braunschweig, Germany, the National Animal Protection Law (Tierschutzgesetz (TierSchG)) and Animal Experiment Regulations (Tierschutz-Versuchstierverordnung (TierSchVersV)) and the recommendations of the Federation of European Laboratory Animal Science Association. The study was approved by the Lower Saxony State Office for Nature, Environment and Consumer Protection (LAVES), Oldenburg, Lower Saxony, Germany (permit number 33.19-42502-04-19/3293 and permit number 33.9-42502-04-21/3795). C57BL/6N SPF-H mice were bred at the animal facilities of the Helmholtz Center for Infection Research (HZI) under enhanced SPF conditions^60^. GF C57BL/6NTac mice and OMM^12^ C57BL/6NTac mice were bred in isolators (Getinge) in the germ-free facility at the HZI. Animals used in experiments were gender and age matched. Animals were randomly assigned to cages. Female and male mice with an age of 8–16 weeks were used. Sterilized food and water were provided ad libitum. Mice were kept under a strict 12 h light cycle (lights on at 7:00 a.m. and off at 7:00 p.m.) and housed in groups of up to six mice per cage. All mice were euthanized by asphyxiation with CO_2_ and cervical dislocation.

#### Bacterial strains

The *S*. Typhimurium SL1344 strain EM12442 harboured a constitutively expressed β-lactamase resistance gene (*bla*) stably integrated at the phage P22 attachment site, which confers ampicillin resistance, and a chromosomally integrated *luxCDABE* cassette, which confers kanamycin resistance^61^. *S*. Typhimurium strain EM12442 was used for all infection experiments and in vitro assays. Furthermore, clinical *S. enterica* strains of different serovars and multilocus sequence types derived from nationwide genomic surveillance of the German National Reference Center for *Salmonella* and other Bacterial Pathogens encompassing ~4,000–5,000 clinical *Salmonella* strains per year were used for ex vivo assays and were obtained from the Robert Koch Institute in Werningerode. *S*. Typhimurium strain EM8317 (wild-type *S*. Typhimurium strain LT2 harbouring pKH70-PrpsM-sfGFP, ApR) was used for microfluidic mother machine experiments (Supplementary Table 1). The *K. oxytoca* isolate (MK01) used for in vivo experiments was obtained from the human cohort generated in a former study^4^. Further KoSC isolates used for ex vivo assays were either isolated from the ongoing human cohort studies at the HZI (MR, MikroResist; MK, MikroKids; LK, LöwenKids) or obtained from the Hannover Medical School (Supplementary Table 1).

### Method details

#### Isolation of commensal KoSC strains from human stool samples

Stool samples were homogenized in 1 ml phosphate-buffered saline (PBS) and plated in serial dilutions on CHROMagar Orientation plates. Blue colonies were picked and plated on Luria–Bertani (LB) agar plates with ampicillin to check for natural ampicillin resistance. Positive colonies were sent for 16S ribosomal RNA gene sequencing and whole-genome sequencing.

#### In vivo infection experiments

SPF mice received ampicillin (0.5 g l^−1^) for 7 consecutive days starting 3 days before colonization with *K. oxytoca*. OMM^12^ and GF mice were naturally susceptible to *S*. Typhimurium and did not receive ampicillin water before colonization. Inocula were prepared by culturing bacteria overnight at 37 °C in LB broth. Subsequently, the culture was diluted 1:25 in fresh medium and subcultured for 4 h at 37 °C in LB broth. Bacteria were resuspended in 10 ml PBS, and the required amounts were calculated. Mice were orally inoculated with 5 × 10^8^ c.f.u. of *K. oxytoca* diluted in 200 µl PBS. After 4 days of precolonization, mice were orally infected with 5 × 10^5^ c.f.u. of *S*. Typhimurium. Bacteria were prepared as described before for *K. oxytoca*. The weight and survival of the mice were monitored daily, and faeces were collected at different time points of the experiment. Depending on the used mouse model, animals were killed on day 2 p.i. (SPF-abx model) or day 6 p.i. (OMM^12^) to evaluate the pathogen burden in the intestinal organs. GF animals were killed at various time points if they reached a critical BWL of more than 20%.

#### Quantification of faecal *K. oxytoca and S*. Typhimurium colony-forming units

Fresh faecal samples were collected, and weight was recorded. Subsequently, faecal samples were diluted in 1 ml PBS and homogenized by bead-beating with 1 mm zirconia/silica beads two times for 25 s each time using a Mini-Beadbeater-96 (BioSpec). To determine colony-forming units, serial dilutions of homogenized samples were plated on LB plates with 50 µg ml^−1^ kanamycin and CHROMagar Orientation plates (blue colonies, *K. oxytoca* MK01/ DSM5175; purple colonies, *E. coli* Mt1b1; white colonies, *S*. Typhimurium). Plates were cultured at 37 °C for 1 day before counting. The colony-forming units of *K. oxytoca and S*. Typhimurium were calculated after normalization to the weight of faeces.

#### Calculation of CI ratio

The CIs were calculated quotients based on the absolute numbers of *S.* Typhimurium and the commensal bacteria (*K. oxytoca*/*E. coli)* after 24 h of co-cultivation in various media. Whether the starting ratio was 1:1 or 1:10 was also taken into consideration. Therefore, in some displayed items an equal ratio (solid line) is displayed at 10^0^ (1:1 ratio) or 10^−1^ (1:10 starting ratio). If the interaction between both species was neutral, the initial relative abundance was maintained. By contrast, CI lower than 10^−^^1^ indicated an inhibitory effect of the commensal bacteria on *S*. Typhimurium growth, while values higher than 10^−1^ reflected a competitive advantage of *S.* Typhimurium over the commensals.

#### Ex vivo assays using caecal content of mice

Mice from different origins (GF, OMM^12^, SPF, SPF-abx) were killed, and caecal content was isolated, weighted and diluted in a 1:1 ratio with PBS and homogenized two times for 25 s each time using a Mini-BeadBeater-96 (BioSpec). Afterward, caecum samples were centrifuged for 10 min at 12,000 × *g*, and the liquid phase was sterile-filtered and used as a media base. Bacteria were grown in LB media and normalized to the required optical density at 600 nm (OD_600_). Wells filled with 225 µl sterile-filtered caecal content were inoculated with 10 µl *S. enterica* (OD_600_ = 0.2) and 20 µl KoSC or *E. coli* strains (OD_600_ = 1) for assays performed with 1:10 pathogen to commensal ratio or inoculated with 10 µl of OD_600_ = 0.2 culture for assays with 1:1 pathogen to commensal ratio and cultivated at 37 °C under aerobic conditions for 24 h. Each sample was serial diluted in 96-well plates and plated on selective LB agar plates with kanamycin (LB-kan agar) and/or CHROMagar to discriminate KoSC (blue colonies) or *E. coli* (pink colonies) from *S*. Typhimurium colonies (white).

#### In vitro assays in defined and semi-defined media

For co-cultivation of *S*. Typhimurium with KoSC strains, the bacteria were grown aerobically in LB (Sigma) and prepared in a 1:10 ratio as described before. Most in vitro assays were performed in a simple TLB with the following composition: 17 g l^−1^ tryptone (Sigma), 10 g l^−1^ lactose (Sigma) and 2.5 g l^−1^ dipotassium hydrogen phosphate, which was previously described to induce toxin production^42^. After 24 h of aerobic incubation at 37 °C, co-cultures were plated on selective LB-kan agar and CHROMagar Orientation plate to detect colony-forming units of *S*. Typhimurium and KoSC. For titration assays, the carbon source lactose was utilized in various concentrations ranging from 2.5 g l^−1^ to 10 g l^−1^. For some assays, the carbon source lactose was exchanged for other sugars in the same proportion of 10 g l^−1^, or other pre-made media were used including BHI (Oxoid), mGAM (HiMedia), LB (Roth) or simple MM9 minimal media supplemented with histidine (0.03%) and an amino acid mix (Gibco). To analyse direct competitive but toxin-independent effects between KoSC and *S*. Typhimurium, bacteria were cultivated in MM9 minimal media with histidine and respective carbon sources in lower concentrations (5 g l^−1^). To do so, each strain was cultivated on R2A agar (Difco) overnight, and bacteria were adjusted to an OD_600_ = 0.2 in MM9 without a carbon source. A 96-well plate was filled with 99 µl of MM9 media with respective carbon sources (5 g l^−1^). About 1 µl of each commensal strain was cultivated together with 1 µl of *S*. Typhimurium for 24 h at 37 °C. After the incubation, colony-forming units were determined as described before.

#### In vitro assays with toxin supplementation

For susceptibility testing against TM and TV, which have been synthesized in Graz according to a previously described protocol^35^, bacteria were cultivated in LB media (*Salmonella* strains and *K. oxytoca*) or BHI (OMM^12^ bacteria) overnight. The next day, bacteria were adjusted to an OD_600_ = 0.2, and 10 µl of each culture was grown in media spiked with various concentrations of TM and TV (20 µM, 40 µM, 80 µM, 160 µM and 320 µM) or solvent (100% EtOH) as a control. The assays were performed under aerobic (enterobacteria) or anaerobic (OMM^12^ bacteria) conditions for 24 h in a 96-well format. OD_600_ was assessed every 30 min, and colony-forming units were determined after 24 h on appropriate agar plates (LB for enterobacteria and BHI–blood plates for OMM^12^ bacteria) as previously described.

#### Phenotypic microarrays

For carbohydrate utilization screenings, the phenotype microarrays PM1 and PM2a from Biolog were used. The array was performed with *K. oxytoca* strain (MK01 WT and sKO) and *S*. Typhimurium as previously described^4^. The Biolog experiments were performed according to the manufacturer’s descriptions with the phenotypic microarray plates PM1 and PM2a, which are 96-well plates coated with lyophilized sugars in the usual range microbes require for growing (exact concentrations are not provided by the manufacturer). One day before the assay was performed, bacteria of interest were streaked on an R2A agar plate (Difco) to deplete the remaining nutrient stores from the cells. The next day, bacteria were resuspended in MM9 media supplemented with 0.03% histidine and adjusted to OD = 0.2 (equals approximately 10^6^ c.f.u. ml^−1^). The bacterial suspensions were then diluted to 1:100 (starting at OD = 0.002), and 100 µl of suspensions was placed in each well. The plates were incubated aerobically in a microplate spectrophotometer with continuous shaking and OD_600_ measurements every 60 min. The obtained values were blanked against well A1, which was the negative control without sugar. The growth over 24 h was displayed as the area under the curve (AUC) as the mean from three independent measurements in a heat map, or colony-forming units were calculated based on the recovered amounts from each well. For the competition assays using Biolog Microarrays, bacteria were streaked on R2A agar and prepared as described before. Then, bacteria were spiked in a 1:1 ratio into each well and grown for 24 h under aerobic conditions before each strain was recovered on selective agar plates (LB-kan for *S.* Typhimurium and CHROMagar Orientation plates for *K. oxytoca*). A growth index was calculated based on the inoculum density in comparison to the final colony-forming units after 24 h. Active growth was defined if at least 2 times more were recovered from a carbohydrate-containing well in comparison to the negative well without a carbon source.

#### Generation of *K. oxytoca* mutants and subsequent complementation

For the construction of *npsA* deletion, stop inserted mutants and double mutants (MK01 sKO*ΔgatABC)*, we utilized a previously adapted CRISPR/Cas9 (clustered regularly interspaced short palindromic repeats and CRISPR-associated protein 9) and lambda-Red recombination-based genome editing protocol^4^^,62,63^. Briefly, *K. oxytoca* was first transformed with the plasmid pCasKP^62^. Expression of Lambda-Red genes was induced with 2% l-arabinose for 2 h. Cells were then co-transformed with a spacer-introduced pSGKP and linear double-stranded DNA assembled from homology arms (500 bp each) neighbouring the deleted or edited gene to serve as a repair template. After overnight incubation at 30 °C on LB plates supplemented with 60 µg ml^−1^ apramycin and 300 µg ml^−1^ spectinomycin, single colonies were screened for the mutant genotype with colony PCR and subsequently with Sanger sequencing. Colonies were cured of both plasmids after overnight incubation at 37 °C in LB medium and, consequently, overnight incubation on LB plates supplemented with 5% sucrose at 37 °C. Ten colonies were checked for apramycin and spectinomycin sensitivity. *K. oxytoca* MK01 genomic DNA was used as a template for the generation of the 500 bp homology arms. Subsequently, the two cassettes were assembled via splicing by overlap extension (SOE)-PCR.

Genetic complementation for the sKO strain was achieved by knocking in the WT gene or gene segment to its original genomic locus. In brief, new spacer sequences were designed to target the bacterial chromosome in the vicinity of the previous genetic edit. Repair templates provided 500 bp homology upstream and downstream from the complementation spacer’s cleavage site. To avoid Cas9-mediated cleavage of the complementation repair template, a single nucleotide substitution was introduced during SOE-PCR assembly of the repair template. The single nucleotide substitution affected the PAM-site of the complementation spacer sequence, changing it from 5′-NGG-3′ to either 5′-NGT-3′ or 5′-NTG-3′ to prevent Cas9-binding and thus cleavage. This leads to specific targeting of the bacterial chromosome, but not the repair template allowing homology-directed repair. The mutation was designed to lead to a synonymous mutation in the coding sequence of the targeted gene leading to no changes in the translated amino acid sequence. Genetic complementation was verified with Sanger sequencing.

#### Scanning electron microscopy

Bacterial cultures were fixed with aldehydes (final concentration 5% formaldehyde and 2% glutaraldehyde) and washed twice in TE buffer (20 mM TRIS and 1 mM EDTA, at pH 6.9). About 50 µl was added to round, poly-l-lysine pretreated coverslips and incubated for 10 min at room temperature followed by a post-fixation in TE buffer including 1% glutaraldehyde for another 10 min. Afterward, coverslips were washed twice in TE buffer and dehydrated on ice in a graded series of acetone (10%, 30%, 50%, 70% and 90%) for 10 min each step, followed by two steps in 100% acetone at room temperature. Critical point drying with liquid CO_2_ was performed with the CPD300 (Leica Microsystems) and sputter coating (55 s at 45 mA) with the SCD500 (Bal-Tec, Liechtenstein) to coat the coverslips (mounted onto aluminium stubs with carbon adhesive discs (Plano, Wetzlar)) with a thin gold–palladium film. Samples were analysed with a field emission scanning electron microscope Zeiss Merlin (Zeiss, Oberkochen) using the Everhart Thornley HESE2 detector and the in-lens SE detector in a 25:75 ratio and with an acceleration voltage of 5 kV. Digital images of calibrated magnifications were acquired with the SmartSEM (version 6.08, Carl Zeiss Microscopy Ltd) software and further analysed with Fiji (2.14.0)^64^ to manually measure the length of horizontally oriented bacterial cells.

#### Microfluidic chip design and fabrication

Microfluidic mother machine devices were fabricated using a soft lithography protocol^65^, which utilizes a master mould for the replication of microchannels in the soft elastomer polydimethylsiloxane (PDMS). A 6′-silicon wafer master mould was fabricated by Electron Beam Lithography (ConScience AB, Sweden) and contains 12 device layouts. The mother machine design used in this study features three cultivation channels with two inlets for switching between different media, as previously described^66^. Each channel contains an array of mother machine traps, organized in 57 blocks with 21 traps in every block. The traps are 30 μm in length, 1 μm in width and 0.8 μm in height, with a 0.3 µm wide constriction at the bottom. The PDMS chips were fabricated by mixing the SYLGARD 184 silicone elastomer base (Dow, Europe) with the curing agent at a 7:1 ratio. The mixture was subsequently degassed under vacuum for 30 min, poured onto the wafer and cured overnight at 80 °C. Upon detaching the cured PDMS from the wafer, individual chips were cut with a scalpel, and inlets and outlets were punched in the chip using a punching tool (Robbins True-Cut Disposable Biopsy Punch 0.75 mm with Plunger, Robbins Instruments). The surface of the chip was cleaned by a rinse with acetone and isopropanol and the application of adhesive tape (tesafilm). The PDMS chip was bonded to a high-precision coverslip (24 × 60 mm) by applying oxygen plasma to both the chip and coverslip surfaces for 60 s at a pressure of 0.8 mbar in a plasma cleaner (Diener Zepto, Diener Electronic). The bond was strengthened by incubation of the bonded device at 80 °C for 5 min. The device was mounted on an inverted fluorescence microscope for time-lapse imaging (Nikon Eclipse Ti2).

#### Spent media production

Pre-cultures of KoSC strains MK01 WT and sKO were prepared by culturing bacteria overnight in LB broth at 37 °C with continuous shaking. The next day, cultures were diluted 1:200 in TLB and grown for 24 h for toxin production. Samples were centrifuged at 10,000 × *g* for 10 min at 4 °C, and the SNs were sterile filtered and used for microfluidic mother machine experiments.

#### Single-cell growth experiment with spent medium

*S*. Typhimurium strain EM8317 (wild-type *S*. Typhimurium strain LT2 harbouring pKH70-PrpsM-sfGFP, ApR) was grown in LB supplemented with 100 μg ml^−1^ ampicillin at 37 °C overnight. The next day, cells were diluted 1:100 in LB, supplemented with 100 μg ml^−1^ ampicillin and grown at 37 °C until the mid-exponential phase. The cell suspension was loaded undiluted in a 1 ml syringe and connected to the upper channel outlet using silicone tubing (Tygon S-54-HL, inner diameter = 0.51 mm, outer diameter = 1.52 mm, VWR International) and blunt dispensing needles (general-purpose tips, inner diameter = 0.41 mm, outer diameter = 0.72 mm, Nordson EFD). By manually perfusing the cell suspension, most of the mother machine traps were loaded with at least one cell. Using this technique, two channels in the microfluidic device were loaded to allow tracking of the cells exposed to the spent media containing TM (from MK01 WT) and the control spent media from MK01 sKO in the same experiment. The channel outlets were connected to waste reservoirs.

Fresh TLB and spent media (from MK01 WT and MK01 sKO) were supplemented with 100 μg ml^−1^ ampicillin and 0.5 mg ml^−1^ of BSA and sterile filtered. Fresh TLB was supplemented with 0.1 μm STAR RED maleimide (Abberior, Germany) to enable visual tracking of the medium switch from fresh TLB to spent media and back to fresh TLB. The fresh and spent media for each channel were stored in individual 50 ml reservoirs (P-CAP series, FLUIGENT), which were connected to the outlets of a programmable pressure regulator (MFCS, FLUIGENT). Hence, each medium in the respective reservoir was pressurized by one individual outlet of the pressure regulator. The media were delivered into the chip via silicone tubing connected to the inlets with blunt dispensing needles. TLB was connected to the upper inlet of the channel, while spent media were connected to the lower inlets. The pressure at the top inlet was set to 300 mbar and was kept constant throughout the experiment. The pressure on the lower inlet was set to 285 mbar at the beginning of the experiment to enable the cultivation of cells on TLB. Cells were kept on TLB for approximately 2 h on the microfluidic device before starting the image acquisition to facilitate adaptation to the environment of the microfluidic device. The supply of TLB was continued for another 3 h after the start of image acquisition. The switch to spent media was activated by a programmed increase of the pressure on the lower inlet to 315 mbar. The pressure on the lower inlets was decreased after another 4 h to 285 mbar to allow TLB again to reach the cells.

#### Time-lapse microscopy

Time-lapse microscopy was carried out using a Nikon Eclipse Ti2 inverted microscope equipped with a CFI Plan Apochromat DM ×60 Lambda NA1.40 Ph3 oil objective, an Orca Fusion BT camera (Hamamatsu), a focus stabilization system (Perfect Focus System, Nikon), a SPECTRA III LED light source (Lumencor) and a temperature-controlled chamber (Okolab). An LED-DA/FI/TR/Cy5/Cy7-A Full Multiband Penta filter (Semrock, IDEX) was used for imaging. The temperature of the chamber was set to 37 °C for the duration of the experiment. Phase contrast and fluorescence were taken every 5 min for 15 h. Images on the phase contrast channel were taken with 100 ms exposure, and a 647 nm LED was used with 5% of the maximum illumination power and 100 ms of exposure.

#### Image analysis

While red fluorescence was used for an exact determination of the media switching events, the phase-contrast imaging channel was utilized for the analysis of cellular growth. We used an in-house written pipeline for the rotation and alignment of 30.tif stacks of individual imaged mother machine blocks. The pipeline utilized basic FIJI^64^ functions for rotation and translation. From the aligned stacks, individual traps were cropped, out and the corresponding frames were arranged next to each other from left to right in increasing order, resulting in kymographs. The kymographs underwent segmentation in Ilastik (v. 1.4.0)^67^. The segmentation result was exported as binary masks in.tif format, where the cells appear white and the surrounding area black. Subsequently, the binary masks were processed in Matlab (v. R2022b) (Mathworks). Each cell was detected as an object by using Matlab’s regionprops function, which returned the relevant data regarding cell geometry and position within the kymograph. Cell length was estimated as the ‘major axis length’ of the regionprops function.

Data analysis requires the cells to be organized in division cycles to extract growth rates. A cell division cycle corresponds to a cell from birth until division, death or escape from the mother machine trap. A decision-making algorithm was implemented in Matlab to link cells to cycles. The algorithm starts by linking the objects at the lowest position of the kymograph into a cycle, going from left to right, thereby following the time sequence frame by frame. When the object’s length at time *t* + 1 was less than 90% of the length at *t*, the algorithm registered a division event, and the cycle terminated. A new cycle for the bottom daughter cell was initiated, and so on. After completion of the bottom cycles, the corresponding objects were deleted from the kymograph mask, and the cycle assignment continued with the objects which now got the lowest positions.

The growth rate was estimated from the slope of a linear fit to the logarithm of cell length over each cell cycle. The minimal duration of a cycle for the calculation of a growth rate corresponds to three frames. Shorter cycles were not considered for growth rate calculation. For cycles that last longer than three frames, the preferred cycle duration to calculate the growth rate was set to four frames. Thereby, very long cycles, as they appear during phases of growth arrest due to exposure to spent media, were subdivided into intervals with a preferred duration of four frames for growth rate calculation.

#### Semi-quantitative scoring of inflammation

Proximal colon samples were fixed in 4% neutrally buffered formaldehyde and embedded in paraffin according to standard histological procedures. Sections of 3 μm thickness were stained with haematoxylin and eosin (HE) for standard scorings and evaluated by a specialist blinded to the experimental groups using light microscopy. The histological scoring used to evaluate the severity of inflammation was modified from the TJL score developed by The Jackson Laboratory^68^. The alteration of the score has been previously described^69^. All sections were scored from 0 to 3 for the general criteria severity, inflammatory infiltrate, villous atrophy, crypt damage and percentage of area involved leading to a maximum score of 15 for very severe inflammation.

#### Semi-quantitative analysis of toxins in in vitro samples

KoSC strains were grown for 24 h in 1 ml tryptone lactose media or 1 ml GF caecum content to induce toxin production. Samples were centrifuged for 10 min at 16,000 × *g*, and the SN was sterile filtered, transferred into glass vials and kept at −80 °C until further processing. Ultra-performance liquid chromatography (LC)–high-resolution mass spectrometry (MS) analysis was performed on a Dionex (Germering) Ultimate 3000 RSLC system using a Waters (Eschborn) BEH C18 column (50 × 2.1 mm, 1.7 µm) equipped with a Waters VanGuard BEH C18 1.7 µm guard column. Separation of 1 µl sample was achieved by a linear gradient from (A) H_2_O + 0.1% FA to (B) ACN + 0.1% FA at a flow rate of 600 µl min^−1^. The column was thermostated at 45 °C. The gradient conditions were as follows: 0–0.5 min, 5% B; 0.5–18.5 min, 5–95% B; 18.5–20.5 min, 95% B; 20.5–21 min, 95–5% B; 21–22.5 min, 5% B. The LC flow was split to 75 µl min^−1^ before entering the Bruker Daltonics timsTOF-fleX/ timsTOF-Pro mass spectrometer (Bremen) equipped with an Apollo II ESI (electrospray ionization) source. Mass spectra were acquired in centroid mode ranging from 150 to 2,500 *m*/*z* at a 2 Hz full scan rate. MS source parameters were set to 500 V as end plate offset, 4,000 V as capillary voltage, nebulizer gas pressure at 1 bar, dry gas flow of 5 l min^−1^ and a dry temperature of 200 °C. Ion transfer and quadrupole settings were set to funnel RF 350 Vpp, multipole RF 400 Vpp as transfer settings and ion energy of 5 eV as well as a low mass cut of 120 *m*/*z*. The collision cell was set to 5.0 eV, and pre-pulse storage time was set to 5 µs. The spectral acquisition rate was set to 2 Hz. Internal calibration was achieved using automatically injected sodium formate solution before every LC–MS run directly into the source and calibration on the respective clusters formed in the ESI source. All MS analyses were recalibrated to sodium formate clusters in the first 0.2 min of the LC run. The abundance of the compounds TM and TV was evaluated using Bruker Compass Metaboscape 2022b version 9.0.1 (Bremen). Features above an intensity of 10,000 counts that appeared in 5 consecutive spectra were annotated. The compounds TM with a retention time of 2.7 min and an *m*/*z* of 234.1004 and TV with a retention time of 6.7 min and an *m*/*z* of 333.1477 were annotated when signals within 5 p.p.m. and 0.2 min retention time were observed.

#### Absolute quantification of toxins in in vitro and in vivo samples

The synthesis of ^15^N-labelled TM and TV used for analyte quantification was described previously^70^. TM and TV were extracted from faeces (or caecum contents or various tryptone media supplemented with sugar) and quantified by high-performance liquid chromatography (HPLC) high-resolution ESI MS as previously described^70^. In brief, samples were collected and stored in HPLC glass vials at −80 °C until further processing. For extraction, pre-weighed samples were homogenized by vortexing. EtOH-dissolved ^15^N-labelled TM (20 µM) and TV (0.2 µM) were added as internal standards to each sample and vortexed again for 5 min. Samples were evaporated to dryness (10 mbar, 40 °C, 60 min), rehydrated in 20 µl water to maximize signal-to-noise ratio and extracted with 200 µl *n*-butanol by vortexing for 5 min. Extracts were centrifuged in glass vials (15 min, 4,000 × *g*, 20 °C), and the organic phase was filtered (0.2 µm, Nylon) and stored at −20 °C until measurement. For each sample set, the lowest measurable TM concentration was used to define the limit of quantification.

#### Quantification of total free carbon sources in caecal contents

For quantification of total free carbohydrates, the ‘Total Carbohydrate Assay Kit’ from Sigma-Aldrich was used (catalogue number MAK104) according to the manufacturer’s protocol. Caecal contents were extracted from GF, OMM^12^, untreated SPF mice and ampicillin-treated SPF mice, aliquoted (approximately 100 mg), diluted with 1 ml of ice-cold assay buffer and homogenized with 1 mm zirconia beads using a minibead-beater 2 times for 25 s each time. Afterward, samples were centrifuged at 13,000 × *g* for 5 min to remove insoluble material. About 5 µl of sample SN were used for measurements. For glucose standard, 0, 2, 4, 6, 8 and 10 µl of the 2 mg ml^−1^ standard solution was pipetted directly into a 96-well plate, generating 0 (blank), 4, 8, 12, 16 and 20 µg per well standards. Water was added to each well to bring to a final volume of 30 µl. Samples were prepared in duplicates. Next, 150 µl of the concentrated sulfuric acid was added. Wells were mixed by pipetting and incubated for 15 min at 90 °C in the dark. Then, 30 µl of developer was added to each well, and the plate was mixed at room temperature for 5 min using a horizontal shaker before the absorbance of contents was measured at 490 nm (*A*_490_). Resulting duplicate values were averaged, blank values were subtracted and resulting concentrations were calculated using the standard curve.

#### Quantification of lactose/galactose in caecal contents

For quantification of lactose/galactose, the ‘Lactose Assay Kit’ from Sigma-Aldrich was used (catalogue number MAK017) according to manufacturer’s protocol. Caecal contents were extracted from GF, OMM^12^, untreated SPF mice and ampicillin-treated SPF mice, aliquoted (approximately 100 mg), diluted in 4 volumes of the lactose assay buffer and homogenized with 1 mm zirconia beads using a minibead-beater 2 times for 25 s each time. Afterward, samples were centrifuged at 13,000 × *g* for 10 min to remove insoluble material. About 50 µl of sample SN was used for measurements. For the lactose standard, 10 ml of the 100 nmol ml^−1^ lactose standard was diluted in 990 ml of lactose assay buffer to generate a 1 nmol ml^−1^ standard solution. About 0, 2, 4, 6, 8 and 10 ml of the 1 nmol ml^−1^ lactose standard was pipetted into a 96-well plate, generating 0 (blank), 2, 4, 6, 8 and 10 nmol per well standards. Lactose assay buffer was added to each well to bring the volume to 50 ml. Samples were prepared in duplicates. Next, 2 µl of the lactase and 50 µl of a prepared master reaction mix (per sample: 44 µl lactose assay buffer, 2 µl probe, 2 µl lactose enzyme mix and 2 µl horseradish peroxidase) were added to each well. Wells were mixed by pipetting and incubated for 60 min at 37 °C in the dark. Then, the absorbance of contents was measured at 570 nm (*A*_570_). Resulting duplicate values were averaged, blank values were subtracted and resulting concentrations were calculated using the standard curve.

#### DNA isolation

Faeces samples were collected and stored at −20 °C until processing for DNA-based 16S rRNA gene sequencing. About 700 µl lysis buffer (Zymo) and 100 µl of zirconia/silica beads (0.1 mm diameter) (Roth) were added per 100 mg faeces sample. Lysis of bacteria was performed by mechanical disruption using a Mini-BeadBeater-96 (BioSpec) three times for 5 min each time, with 5 min on ice in between. After centrifugation, 200 µl of SN was transferred into a 96-deep well plate (Nunc) and used for purification. DNA was extracted using the Zymo MagKit 96 well according to the manufacturer’s instructions with 40 µl beads on a Tecan Fluent.

#### RNA isolation and complementary DNA synthesis

Bacteria were grown for 8 h in tryptone media with or without added galactose or lactose (10 g l^−1^) or in sterile filtered caecal content of mice. Equal amounts of RNAshield (Zymo Research) were added per sample. Samples were incubated for 15 min at room temperature and vortexed every 5 min. Afterward, samples were centrifuged for 5 min at 8,000 × *g*, and pellets were stored at −80 °C until further processing. Total RNA was extracted from bacterial pellets using the ZymoBiomics RNA Miniprep Kit (Zymo Research) according to the manufacturer’s descriptions. Crude RNA extract was treated with DNAse to remove the remaining DNA. The obtained total RNA was checked for quality and concentration using DenoVix Spectrophotometer (Biozym). Next, 2 µg of total RNA was used as input for complementary DNA synthesis using 200 U μl^−1^ RevertAid Reverse Transcriptase (Thermo Scientific). cDNA was diluted 1:10 and used for subsequent quantitative PCR.

#### Quantitative PCR

Quantitative real-time PCR was performed in a CFX96 Real-Time PCR detection system (BioRad) to quantify the gene expression levels of *npsA* in relation to *mdh* as a housekeeping gene. The following primer sequences were used: npsA_1_for GTGGTGTCGGGAGACTTTGT, npsA_1_rev ACCACCATCTCAACCAGAGG, mdh_1_f GCGTCGGGATTATCACCAAC and mdh_1_r CCTTTCAGTTCCGCCACAAA. For quantitative PCR reactions, a master mix of the following components was prepared: 0.5 μl (10 μM) of forward primer, 0.5 μl (10 μM) of reverse primer, 5 μl of 2× SybrFast Master Mix and 4.5 μl of cDNA (~100 ng). Amplification was performed in triplicate wells for each sample. In each set of reactions, *mdh* was used as a reference gene for the normalization of the *npsA* cDNA amount. Real-time PCR analysis was performed using the following assay conditions: (1) pre-incubation (95 °C for 1 min); amplification and quantification programs were repeated for 40 cycles (95 °C for 3 s, 59 °C for 30 s, 72 °C for 10 s with a single fluorescence measurement), (2) melting curve program (95 °C for 10 s, 65 °C for 1 min with continuous fluorescence measurement at 95 °C) and (3) a cooling step at 40 °C for 10 s. The relative gene expression was calculated using the 2^−ΔΔCt^ method^71^.

#### 16S rRNA gene amplification and sequencing

16S rRNA gene amplification of the V4 region (F515_GTGCCAGCMGCCGCGGTAA/R806_ GGACTACHVGGGTWTCTAAT) was performed according to an established protocol previously described^72^. Briefly, DNA was normalized to 25 ng µl^−1^ and used for sequencing PCR with unique 12-base Golary barcodes incorporated via specific primers (obtained from Sigma). PCR was performed using Q5 polymerase (NewEnglandBiolabs) in triplicates for each sample, using PCR conditions of initial denaturation for 30 s at 98 °C, followed by 25 cycles (10 s at 98 °C, 20 s at 55 °C and 20 s at 72 °C). After pooling and normalization to 10 nM, PCR amplicons were sequenced on an Illumina MiSeq platform via 300 bp paired-end sequencing (PE300). Using the Usearch (8.1) software package (http://www.drive5.com/usearch/) (v.11.0.667) the resulting reads were assembled, filtered and clustered. Sequences were filtered for low-quality reads and binned based on sample-specific barcodes using QIIME (v.1.8.0)^73^. Merging was performed using -fastq_mergepairs—with fastq_maxdiffs 30. Quality filtering was conducted with fastq_filter (-fastq_maxee 1), using a minimum read length of 300 bp and a minimum number of reads per sample = 1,000. Reads were clustered into 97% identify operational taxonomic units (OTUs) by de novo OTU picking, and representative sequences were determined by use of the UPARSE algorithm of Usearch^74^. Abundance filtering (OTUs cluster >0.5%) and taxonomic classification were performed using the Ribosomal Database Project (RDP) Classifier (naive Bayesian classifier v.2.10.19) executed at 80% bootstrap confidence cut-off^75^. Sequences without matching reference datasets were assembled as de novo using UCLUST within Usearch. Phylogenetic relationships between OTUs were determined using FastTree to the PyNAST alignment^76^. The resulting OTU absolute abundance table and mapping file were used for statistical analyses and data visualization in the R statistical programming environment package phyloseq^77^. It is noteworthy that, as the V4 region is nearly identical for *Klebsiella* and *Salmonella*, reads were assigned to each species using an open-reference protocol using the 16S rRNA V4 gene regions of *S. enterica* (NR074799), *K. oxytoca* (AF129440) and the OligoMM strains implemented in QIIME (ref. ^72^).

#### Whole-genome sequencing

To assess the taxonomy of all KoSC isolates, bacteria were processed for whole-genome sequencing. First, genomic DNA was extracted using the ZymoBIOMICS 96 MagBead DNA Kit according to the manufacturer’s instructions. Afterward, libraries of each isolate were prepared using the Illumina DNA PCR-Free Prep and quantified with the KAPA Library Quantification Kit Illumina Platforms. Samples were pooled and sent for whole-genome sequencing performed by the group of genome analysis at Helmholtz-HZI Center for Infection Research using NovaSeq 6000 S4 Reagent Kit v1.5 (300 cycles) and targeting depth of 1 million reads per sample. The resulting KoSC genomes have been deposited in GenBank under accession code PRJEB61973.

#### Statistical analysis

The collection of human faeces samples was randomized and blinded. Histological analysis was performed blinded. No other data collection and analysis was performed blind to the conditions of the experiments. No statistical methods were used to pre-determine sample sizes, but our sample sizes are similar to those reported in previous publications^4,8^. No animals or data points were excluded from the analyses. Data distribution was assumed to be normal, but this was not formally tested. Experimental results were analysed using Fiji (2.14.0), GraphPad Prism (v9.1), Matlab (9.14), Usearch (8.1), R studio (4.3.1) with packages phyloseq (1.46.0), ggplot2 (3.4.4), scales (1.3.0.), plyr (1.8.9), ape (5.7.1.), knitr (1.45) and Microsoft Excel (2016). *P* values indicated were analysed by two-sided Mann–Whitney *U*-test, Kruskal–Wallis test (for two group comparisons), log-rank test (for survival curve analysis) or one-way analysis of variance (ANOVA) (multiple groups) with various post hoc tests (Dunn’s, Holm-Šídák’s, Tukey’s) as indicated in each figure legend. No specific adjustments were made for multiple comparisons as the applied post hoc tests specifically account for multiple comparisons and maintain alpha at the specified level (0.05). For 16S rRNA gene sequencing data analysis, OTUs with Kruskal–Wallis test <0.05 were considered for analysis. *P* values lower than 0.05 were considered significant: *P < 0.05, **P < 0.01, ***P < 0.001, ****P < 0.0001^78^. Description of each statistical test and exact *P* values are provided in the source data tables for each figure item.

### Reporting summary

Further information on research design is available in the Nature Portfolio Reporting Summary linked to this article.

### Supplementary information

Reporting Summary

Supplementary Video 1 Visualization of *S.* Typhimurium in the presence of toxin-containing supernatant of *K. oxytoca* MK01 WT.

Supplementary Video 2 Visualization of *S.* Typhimurium in the presence of non-toxin-containing supernatant of *K. oxytoca* MK01 sKO.

Supplementary Table 1 Supplementary information on all strains used in this study.

### Source data

Source Data Figs. 1–6 and Source Data Extended Data Figs. 1–10 Unprocessed raw data and statistical analysis data.

## Data Availability

16S rRNA gene sequencing and bacterial strain genome sequencing data are available at ENA (European Nucleotide Archive) and GenBank under the accession number PRJEB61973. Raw data can be found in the source data files for each figure item. Source data are provided with this paper.

## Code availability

The script for the quantification of growth phenotypes in the mother machine experiments is available via GitHub at https://github.com/SalmoLab/MotherMachine_NatMicro2024 and via Zenodo at 10.5281/zenodo.10974764 (ref. ^79^).
