# Supplementary material for: Klebsiella oxytoca inhibits Salmonella infection through multiple microbiota-context-dependent mechanisms
Source: Nat Microbiol. 2024 Jun 11;9(7):1792–811. doi: 10.1038/s41564-024-01710-0 (PMC11222139; doi:10.1038/s41564-024-01710-0)
Supplement: Supplementary file 1 — Reporting Summary [file 41564_2024_1710_MOESM1_ESM.pdf]

nature portfolio

Corresponding author(s): Till Strowig

Last updated by author(s): 15.03.2024

Reporting Summary

Nature Portfolio wishes to improve the reproducibility of the work that we publish. This form provides structure for consistency and transparency in reporting. For further information on Nature Portfolio policies, see our Editorial Policies and the Editorial Policy Checklist.

Statistics

For all statistical analyses, confirm that the following items are present in the figure legend, table legend, main text, or Methods section.

n/a	Confirmed	
☐	☒	The exact sample size (*n*) for each experimental group/condition, given as a discrete number and unit of measurement
☐	☒	A statement on whether measurements were taken from distinct samples or whether the same sample was measured repeatedly
☐	☒	The statistical test(s) used AND whether they are one- or two-sided<br>*Only common tests should be described solely by name; describe more complex techniques in the Methods section.*
☒	☐	A description of all covariates tested
☐	☒	A description of any assumptions or corrections, such as tests of normality and adjustment for multiple comparisons
☐	☒	A full description of the statistical parameters including central tendency (e.g. means) or other basic estimates (e.g. regression coefficient) AND variation (e.g. standard deviation) or associated estimates of uncertainty (e.g. confidence intervals)
☐	☒	For null hypothesis testing, the test statistic (e.g. *F*, *t*, *r*) with confidence intervals, effect sizes, degrees of freedom and *P* value noted<br>*Give P values as exact values whenever suitable.*
☒	☐	For Bayesian analysis, information on the choice of priors and Markov chain Monte Carlo settings
☒	☐	For hierarchical and complex designs, identification of the appropriate level for tests and full reporting of outcomes
☒	☐	Estimates of effect sizes (e.g. Cohen's *d*, Pearson's *r*), indicating how they were calculated

Our web collection on statistics for biologists contains articles on many of the points above.

Software and code

Policy information about availability of computer code

Data collection	SmartSEM software (6.08, Carl Zeiss Microscopy Ltd)
Data analysis	GraphPad Prism (v9.1), Matlab (9.14), Usearch (8.1), R studio (4.3.1) with packages phyloseq (1.46.0),ggplot2 (3.4.4), scales version (1.3.0.), plyr (1.8.9), ape (5.7.1.), knitr (1.45), Microsoft office (2016), Adobe Illustrator (2023)<br>Image alignment, rotation, and Kymograph plots were created with FIJI/ImageJ (Version: ImageJ 2.14.0/1.54f) 2. Segmentation of Kymographs was performed in Ilastik (Version: 1.4.0) using the Segmentation Workflow “Pixel Classification”<br>Cell division cycle generation and data plots were performed in Matlab (Version R2022b)

For manuscripts utilizing custom algorithms or software that are central to the research but not yet described in published literature, software must be made available to editors and reviewers. We strongly encourage code deposition in a community repository (e.g. GitHub). See the Nature Portfolio guidelines for submitting code & software for further information.

Data

Policy information about availability of data

All manuscripts must include a data availability statement. This statement should provide the following information, where applicable:

- Accession codes, unique identifiers, or web links for publicly available datasets
- A description of any restrictions on data availability
- For clinical datasets or third party data, please ensure that the statement adheres to our policy

16S rRNA gene sequencing and bacterial strain genome sequencing data have been deposited in the ENA (European Nucleotide Archive) under the accession number: PRJEB61 973. Raw data can be obtained from the source data files. Code for mother machine experiment is available on request from Marc Erhardt

Research involving human participants, their data, or biological material

Policy information about studies with human participants or human data. See also policy information about sex, gender (identity/presentation), and sexual orientation and race, ethnicity and racism.

Reporting on sex and gender	Fecal samples from the Löwenkids, MikroKids and MikroResist cohort were obtained from healthy german adults and children. Information on age were collected and are listed in the supplementary table S1. No other metadata were collected during this study. The isolates from the hospital environment were obtained from patients of the Hospital in Hannover (MHH). Only Age and origin of the isolate was documented and can be retrieved from supplementary table S1. The sex and gender of patients with bacterial isolates was not included and reported in the study.
Reporting on race, ethnicity, or other socially relevant groupings	Socioeconomic status was not reported. Race, ethnicity or other socially relevant parameters were also not reported in the study, since they were not part of the analysis.
Population characteristics	Patients or patient characteristics were not part of the analysis and no patient data are reported. We only analyzed bacterial isolates, which were selected based on the following criteria: strain, antibiotic resistance, isolation from patients, no environmental bacteria. Bacterial isolates fulfilling the relevant criteria were stored at the Institute of Medical Microbiology and Hospital Epidemiology. Pseudonymisized bacterial strains were used for analysis.
Recruitment	Participants were recruited via personal contact and an email news-letter for the healthy study participants. No active recruitment was performed for the hospital isolates as this was part of the routine diagnostic screening of patients.
Ethics oversight	Ethics Committee Lower Saxony (permit No. 8629_BO_K_2019; No. 8750_BO_K_2019 and 10392_BO_K_2022)

Note that full information on the approval of the study protocol must also be provided in the manuscript.

Field-specific reporting

Please select the one below that is the best fit for your research. If you are not sure, read the appropriate sections before making your selection.

☒ Life sciences ☐ Behavioural & social sciences ☐ Ecological, evolutionary & environmental sciences

For a reference copy of the document with all sections, see nature.com/documents/nr-reporting-summary-flat.pdf

Life sciences study design

All studies must disclose on these points even when the disclosure is negative.

Sample size	No statistical methods were used to pre-determine the sample size. For animal studies, sample size was chosen according to institutional directives and in accordance with the 3Rs rules (Replacement, Reduction and Refinement) guiding principles underpinning the humane use of animals in research. The necessary number of animals was estimated based on the required numbers reported in previous studies (Osbelt et al., Cell Host and Microbe 2021, Osbelt et al., Plos Pathogens 2020).Experiments were designed and performed sequentially to allow adjustment of sample size in follow-up experiments. At least two independent experiments werde performed with at least 3 animals per group. Growth curves, ex vivo assays and measurments of bacterial cells under various growth conditions were always performed with n>3. Sample sizes were chosen pased on previous publications using similar reagents and experimental setups (e.g. Kienesberger et al., Nature Microbiology, 2022; Osbelt et al., Cell Host Microbe, 2021). Sample sizes are indicated in all figure legends. Non-normal distribution was assumed but never formally tested. Testing for statistical significance always employed two-tailed tests if not stated otherwise.
Data exclusions	No data were excluded from the study.
Replication	All attempts at replication were successful, with multiple mice in each group (see sample size above). All experiments were done at least twice, if feasible, each with multiple technical replicates. The exact number of repeats and technical replicates are listed in each figure legend.
Randomization	Mice were randomly allocated to different treatments. We ensured that in each experiment all mice were siblings and shared the same cage for microbiome homogenization prior to the experiment.

Blinding	Human stool samples were randomized and blinded. For animal studies the investigators were not blinded to group allocation as blinding does not apply to this study because the investigators needed to identify the cages of mice for subsequent colonization resistance tests. Histological examination was blinded. For the other experiments no blinding was performed. This is common as the types of experiments conducted needs knowledge of strain identity/treatments by the investigators.

Reporting for specific materials, systems and methods

We require information from authors about some types of materials, experimental systems and methods used in many studies. Here, indicate whether each material, system or method listed is relevant to your study. If you are not sure if a list item applies to your research, read the appropriate section before selecting a response.

Materials & experimental systems

n/a	Involved in the study
☒	☐ Antibodies
☒	☐ Eukaryotic cell lines
☒	☐ Palaeontology and archaeology
☐	☒ Animals and other organisms
☒	☐ Clinical data
☒	☐ Dual use research of concern
☒	☐ Plants

Methods

n/a	Involved in the study
☒	☐ ChIP-seq
☒	☐ Flow cytometry
☒	☐ MRI-based neuroimaging

Animals and other research organisms

Policy information about studies involving animals; ARRIVE guidelines recommended for reporting animal research, and Sex and Gender in Research

Laboratory animals	(Mus musculus) C57BL/6N SPF-H mice were bred at the animal facilities of the Helmholtz Center for Infection Research (HZI) under enhanced specific pathogen-free (SPF) conditions. Germ-free C57BL/6NTac mice and OMM12 C57BL/6NTac mice were bred in isolators in the germ-free facility at the HZI. Animals used in experiments were gender and age-matched. Animals were randomly assigned to cages. Female and male mice with an age of 8-16 weeks were used. Sterilized food and water ad libitum were provided. Mice were kept under a strict 12-hour light cycle (lights on at 7:00 am and off at 7:00 pm) and housed in groups of up to six mice per cage. All mice were euthanized by asphyxiation with CO2 and cervical dislocation.
Wild animals	The study did not involve wild animals
Reporting on sex	For all animal experiments, groups were age- and gender-matched.
Field-collected samples	This study did not involve samples collected from the field.
Ethics oversight	The study was approved by the Lower Saxony State Office for Nature, Environment and Consumer Protection (LAVES), Oldenburg, Lower Saxony, Germany; permit No. 33.19-42502-04-19/3293 and permit No. 33.9-42502-04-21/3795).

Note that full information on the approval of the study protocol must also be provided in the manuscript.
